# Identification of genetic modifiers of autosomal dominant Alzheimer’s disease: a genome-wide association study

**DOI:** 10.1016/S1474-4422(26)00123-7

**Published:** 2026-06

**Authors:** Maulikkumar Patel, Wei Feng, Nicole S Mckay, Peter R Millar, Menghan Liu, Chengran Yang, Arda Cetin, Matthew Johnson, John Budde, Daniel Western, Thomas W Marsh, Ibrahim O Saliu, Brian A Gordon, Jorge J Llibre Guerra, John C Morris, Randall J Bateman, Eric McDade, David M Holtzman, Natalie S Ryan, Tammie L S Benzinger, Alan E Renton, Alison M Goate, Laura Ibanez, Yun Ju Sung, Guoyan Zhao, Carlos Cruchaga, Cyril Pottier, Dominantly Inherited Alzheimer Network

**Affiliations:** Department of Psychiatry (M Patel PhD, M Liu MS, C Yang PhD, A Cetin MS, M Johnson MS, J Budde MS, D Western PhD, T W Marsh MS, L Ibanez PhD, Prof Y J Sung PhD, Prof C Cruchaga PhD, C Pottier PhD), Neurogenomics and Informatics Center (M Patel, M Liu, C Yang, A Cetin, M Johnson, J Budde, D Western, T W Marsh, L Ibanez, Prof Y J Sung, Prof C Cruchaga, C Pottier), Department of Genetics (W Feng PhD, I O Saliu PhD, G Zhao PhD), Department of Radiology (N S Mckay PhD, B A Gordon PhD, T L S Benzinger MD), Department of Neurology (P R Millar PhD, J J Llibre Guerra MD, Prof J C Morris MD, Prof R J Bateman MD, E McDade MD, Prof D M Holtzman MD, G Zhao, Prof C Cruchaga, C Pottier), Hope Center for Neurological Disorders (Prof J C Morris, Prof D M Holtzman, Prof T L S Benzinger, Prof C Cruchaga), Knight Alzheimer Disease Research Center (Prof J C Morris, Prof D M Holtzman, Prof C Cruchaga), Division of Biostatistics (Prof Y J Sung), and Department of Pathology and Immunology (G Zhao), Washington University School of Medicine, St Louis, MO, USA; Dementia Research Center and UK Dementia Research Institute, University College London Queen Square Institute of Neurology, London, UK (N S Ryan PhD); Ronald M Loeb Center for Alzheimer’s Disease, Department of Genetics and Genomic Sciences, and Nash Family Department of Neuroscience, Icahn School of Medicine at Mount Sinai, New York, NY, USA (A E Renton PhD, Prof A M Goate PhD)

## Abstract

**Background:**

Individuals with autosomal dominant Alzheimer’s disease (ADAD) arising from mutations in *PSEN1*, *PSEN2*, or *APP* exhibit variability in clinical presentation. Genetic studies of ADAD have shaped our understanding of the disease, and the discovery of genetic modifiers can inform therapeutic interventions and improve patient outcomes. We aimed to discover new genetic modifiers in individuals with mutations in the three ADAD genes.

**Methods:**

In this genome-wide association study, we analysed data from participants in three study cohorts (the Knight Alzheimer Disease Research Center [Knight-ADRC], the Dominantly Inherited Alzheimer Network [DIAN] observational study, and the Alzheimer Disease Sequencing Project [ADSP] R4). We did whole-genome sequencing on 101 unrelated, non-Hispanic, White, symptomatic participants with ADAD mutations and 5050 asymptomatic, unrelated control participants. Sensitivity analyses included related participants (148 cases and 5813 controls). We assessed the molecular mechanisms associated with each risk variant, including cis-regulatory effects, plasma protein levels (Knight-ADRC, 2338 participants), CSF concentrations of Alzheimer’s disease biomarkers (DIAN, 64 participants), and neuroimaging data (MRI and PET; DIAN, 64 participants). We evaluated the association of risk variants with age at onset in ADAD and in 6177 participants with sporadic Alzheimer’s disease (ADSP R5).

**Findings:**

Three genome-wide loci with significant risk were associated with ADAD risk, irrespective of the specific ADAD gene mutation. The *CNIH4* locus association was driven by a missense variant (is caused by Gly54Ser, p<0·0001, odds ratio [OR] 11·99 [5·39–26·64]). The *CCNG1* locus risk allele increased the risk of Alzheimer’s disease (p<0·0001, OR 9·56 [4·29–21·24]) and reduced the age at dementia onset (p=0·0068, β=−10·15 [95% CI −17·31 to −2·77]). This allele was also positively associated with Tar DNA binding protein 43 (TDP-43) plasma protein levels and a larger gap between chronological age and structural MRI predicted brain age. The *RHOJ* risk allele (p<0·0001, OR 5·96 [3·42–10·36]) was associated with increased the risk of Alzheimer’s disease, higher CSF total tau (p=0·0056, β=358·37) and phosphorated tau 181 (pTau181; p=0·0006, β=81·28), and lower Aβ42/Aβ40 ratio (p=0·016, β=−0·11) in DIAN ADAD participants, comparing those carrying the risk allele with those not carrying it.

**Interpretation:**

Our findings provide potential insights into disease biology, emphasising the role of Aβ, tau, TDP-43, astrocytes, and angiogenesis in Alzheimer’s disease aetiology. This study offers invaluable insight for family genetic counselling and future clinical trial designs.

**Funding:**

National Institute of Health, National Institute on Aging, Alzheimer’s Association, Hope Center Pilot 2025 Award, NGI Pilot Grant 2025 Award, BrightFocus Foundation, UK Dementia Research Institute at University College London, UK National Institutes for Health and Care Research University College London Hospitals Biomedical Research Centre, Dominantly Inherited Alzheimer Network, Freedom Together Foundation.

## Introduction

Alzheimer’s disease is the most prevalent neurodegenerative disorder, currently ranked as the seventh leading cause of death in the USA. A subset of individuals have Alzheimer’s disease from autosomal dominant mutations in *APP*, *PSEN1*, and *PSEN2* genes, known as autosomal dominant Alzheimer’s disease (ADAD).^1^ These mutations alter the processing of the amyloid precursor protein, increasing the Aβ42 to Aβ40 ratio and promoting the extracellular aggregation of amyloid β into plaques, one of the neuropathological hallmarks of Alzheimer’s disease.^1–3^

Genetic studies of ADAD have shaped our understanding of the disease and have initiated the generation of cellular models, animal models, and therapeutic avenues.^4^ Ryan and colleagues unveiled variability in age at onset (AAO) among carriers of *PSEN1, PSEN2*, and *APP* mutations.^5^ This variability persists even among individuals harbouring identical mutations and within the same family. Identification of factors driving this variability is crucial for genetic counselling and development of precise therapeutic interventions.

Extensive research has explored large families of *PSEN1* mutations, leading to the discovery of multiple candidate genetic modifiers—such as the rare *APOE* Christchurch variant, which potentially delays the AAO by 5 years with one copy and more than 25 years with two copies, and the rare *RELN* variant, which potentially delays the AAO by 20 years.^6–10^ Lee and colleagues reported 37 candidate risk variants requiring further replication in a subset of Carribean Hispanic families with the mutation *PSEN1*_Gly206Ala_.^11^ A fibronectin variant was identified as a protective factor in *APOE* ε*4* homozygous participants.^12^ Altogether, genetic factors involved in key pathological processes such as amyloid deposition, neuronal resilience, and synaptic function underlie the clinical variability observed in individuals carrying ADAD mutations. Although previous research studies on phenotypic modifiers have shown genetic variants associated with specific mutations, additional studies assessing common variants with disease risk across ADAD genes are necessary.^11,13–16^

In this study, we aimed to identify new genetic modifiers in individuals with mutations in the three ADAD genes. We used an unbiased case–control approach, similar to the one used in the discovery of the frontotemporal dementia genetic modifier *TMEM106B*.^17^

## Methods

### Study design and participants

In this genome-wide association study (GWAS), we analysed data from individuals carrying ADAD mutations and control participants from three cohorts— the Knight Alzheimer Disease Research Center (Knight-ADRC),^18^ the Dominantly Inherited Alzheimer Network (DIAN) observational study, and the Alzheimer Disease Sequencing Project (ADSP) R4. Case participants were symptomatic carriers of pathogenic or likely pathogenic ADAD mutations (Clinical Dementia Rating >0·5), whereas control participants were free of neurodegenerative diseases (Clinical Dementia Rating of 0 and non-carriers of ADAD mutations). The list of mutations included in the study is shown in appendix 2 (pp 20–22). Detailed information on participant selection is provided in appendix 2 (p 3).

This study was approved by the Institutional Review Board of Washington University School of Medicine at Saint Louis (202503116). Written informed consent was obtained from all participants or their authorised representatives at each contributing site for Knight-ADRC, DIAN, and the various cohorts comprising the ADSP, with all protocols approved by their respective institutional review boards.

### Sample processing, genotyping, and quality assessment

We included 1992 participants from Knight-ADRC and 472 participants from DIAN. Whole-genome sequencing details are provided in appendix 2 (p 3). We merged the ADSP R4 dataset (16 549 participants) with Knight-ADRC and DIAN, totalling 19 013 participants with 23 696 524 variants. After application of quality control steps to the genotype data to ensure accuracy of variant calls and comparability across cohorts, we identified 101 unrelated non-Hispanic White individuals with ADAD and 5050 control participants with no overlapping participants between cohorts; this cohort is thereafter defined as the discovery cohort (table 1, appendix 2 pp 8–10). We did not identify *APP*, *PSEN1*, and *PSEN2* pathogenic or likely pathogenic variants in our control dataset. Sensitivity analysis included all non-Hispanic White participants, consisting of 148 with ADAD and 5813 control participants (appendix 2 p 23). The detailed methodology is provided in appendix 2 (p 3).

### Genome-wide association analysis

We assessed the association between single nucleotide variants (SNVs, cohort minor allele frequency [MAF_cohort_] ≥0·005) and disease status by fitting a logistic Firth regression model using an additive model in Plink (version 2.0), adjusting for sex, cohort, and the first ten principal components. To assess potential independent signals, we did a stepwise conditional logistic regression analysis for each genome-wide sentinel variant (the most statistically significant SNV that represents an independent trait-associated locus), adjusting for the same covariates. This approach allowed us to control for the effect of the most significant variant to detect potential independent association signals from other variants within the same locus. We also did the GWAS analysis incorporating *APOE* ε*4* as a covariate in the model to assess its impact on the association of sentinel variants with diseases status. To determine the effect of sex, we additionally carried out sex-stratified analyses for the sentinel variants. To leverage genetics information from the related individuals, we also did sensitivity analyses on the sentinel variants (p<5·0 × 10^−8^) using the R package SAIGE with related cases and corrected for unbalanced case–control proportion (appendix 2 p 23). To assess the effect of the sentinel variants per ADAD gene and their interaction with *APOE* ε*4*, we stratified our analysis per gene (appendix 2 p 7) and per gene with *APOE* ε*4* as an interaction term with the sentinel variants, and sex, principal components 1–10, and cohort as covariates. Furthermore, we did a replication analysis for each sentinel variant using non-Hispanic White participants from the ADSP R5 dataset, consisting of 34 individuals with ADAD and 8249 control participants with no overlap with the discovery participants, and did a case-control analysis adjusting for sex and principal components 1–10 (appendix 2 p 30). We did an inverse variance weighted meta-analysis using METAL software, integrating summary statistics from both the discovery and replication datasets under a fixed-effects model. Additionally, we did a case–control analysis on the ADSP R4 participants (11 286 non-Hispanic White participants) to determine whether the identified sentinel variants are associated with sporadic Alzheimer’s disease. We carried out burden tests at the four loci with a minor allele frequency (MAF) of less than 1%, using the SKAT R package and the SKAT-binary function adjusting for sex, cohort, and principal components 1–10.

### Protein quantitative trait loci analysis

We used previously generated plasma proteomics data from 2338 participants in the Knight-ADRC cohort, quantified by SomaScan 7K assay, to assess the association between sentinel variants and protein levels using a linear regression model. Additionally, we assessed the association of the sentinel variants with plasma Tar DNA binding protein 43 (TDP-43) protein concentrations in the Knight-ADRC cohort (2123 participants) using the NULISA platform (Alamar Bio, Riyadh, Saudi Arabia), correcting for age and sex. Only Knight-ADRC samples were assessed by NULISA for TDP-43 concentrations because we did not have DIAN participant-level data (appendix 2 pp 4–5).

### Identification of cis-regulatory effects and chromatin accessibility

To evaluate the effect of the sentinel variants on gene regulation, we analysed computationally predicted and experimentally defined cis-regulatory modules, combined with expression quantitative trait loci (QTL) information and single-nucleus RNA sequencing datasets. Details of the databases, methods, and cutoffs used in the analysis are provided in appendix 2 (pp 5–6).

### Age at onset analysis

We assessed the association between the sentinel variants and AAO of dementia in unrelated participants using linear regression in Plink (version 2.0), adjusting for sex, principal components 1–10, and cohort. We did additional AAO analyses stratifying by sex or including *APOE* ε*4* status as a covariate to evaluate their impact on the association between the sentinel variants and AAO. We then plotted the Kaplan–Meier AAO of dementia survival curves on unrelated participants with ADAD and carried out a log-rank test to assess their differences using the survival (version 3.8–3) and survminer (version 0.5.0) packages in R. To account for relatedness and mutation, we ran a Cox regression incorporating related participants with ADAD adjusting for family identity (appendix 2 p 6). Furthermore, we conducted segregation analyses with AAO of dementia using available clinical and familial data (appendix 2 p 6).

### CSF biomarker analysis

We assessed CSF biomarkers in 64 participants with ADAD in the DIAN cohort (60 with total Tau, 63 with pTau181, and 64 with Aβ42/Aβ40) using the chemiluminescent-based Lumipulse assay.^19^ We did linear regression analyses to assess the effect of the sentinel variants on biomarker levels, adjusting for sex, principal components 1–10, and family identity. We used the lme4 R (version 4.3.2) statistical package for linear regression.

### Neuroimaging analyses

We analysed already available neuroimaging data from 64 related participants of European descent with ADAD. Pittsburgh Compound B PET was used to obtain amyloid burden measurements on 61 participants, quantifying results with partial volume-corrected standardised uptake value ratios in the grey matter of both the right and left precuneus, as defined by FreeSurfer. Fluorodeoxyglucose PET was used to assess the whole-brain cerebral metabolic rate of glucose in 59 participants. Structural MRI acquisitions were analysed in 63 participants to obtain hippocampal volume, in 63 participants to obtain cortical thickness, and in 56 participants for brain age gap. Brain age gap was calculated as the difference between a participant’s chronological age and the estimated age of their brain based on structural MRI data, and is considered a general estimate of brain health or atrophy. To examine the relationships among these measures and sentinel variants, we ran a linear regression adjusting for sex, age at visit (except for brain age gap, which was residualised for age in an earlier step), and family identity (appendix 2 pp 6–7).

### Role of the funding source

The study funders had no role in data collection, data analysis, data interpretation, writing of the manuscript, or the decision to submit for publication.

## Results

We included 1992 participants from Knight-ADRC, 472 from DIAN, and 16 549 from ADSP R4 (control participants and participants with ADAD). We identified 418 individuals (248 symptomatic and 170 asymptomatic) with pathogenic or likely pathogenic mutations in *APP*, *PSEN1*, and *PSEN2*, and 18 595 control participants. After quality control, we selected 5961 non-Hispanic White participants, including 148 participants carrying symptomatic mutations and 5813 control participants. We further refined this to 5151 unrelated non-Hispanic White participants, comprising 101 symptomatic individuals (15 *APP*, 79 *PSEN1*, and seven *PSEN2*; table 1) and 5050 control participants, which constituted the discovery cohort. The median AAO for individuals carrying ADAD mutations was significantly younger (47 years [IQR 40–55, range 35–88]) compared with the control median age at last healthy visit (80 years [IQR 73–86], p<0·0001; table 1). ‘The median AAO was 46 years (38–54) for individuals carrying *PSEN1* mutations, 50 years (46–55) for those carrying *PSEN2* mutations, and 48 years (43–55) for those carrying *APP* mutations. Female participants represented 46 (46%) of 101 case participants and 3019 (60%) of 5050 control participants. The *APOE* ε*4* allele was present in 34 (34%) case participants and 1411 (28%) control participants.

**Figure 1:**
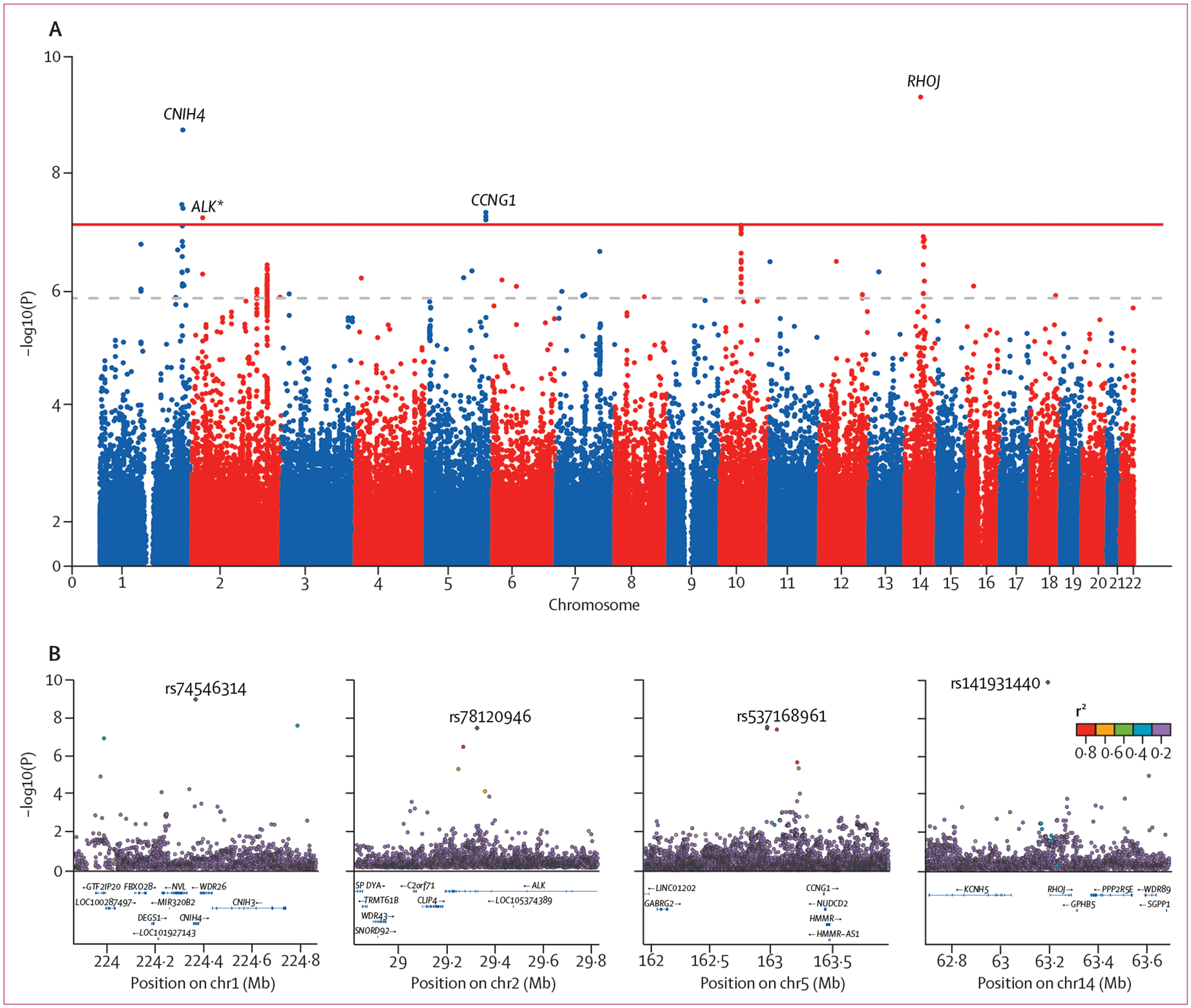
Manhattan and regional plots of the genome-wide association study The association study was done using Firth logistic regression adjusted for relevant covariates. (A) Manhattan plot highlighting the association results per variants according to their genomic position and −log_10_ transformed p values. Each dot represents a SNV, the red plain line represents genome-wide significant thresholds (p=5 × 10^−8^), and the grey dashed line represents genome-wide suggestive association (p=1 × 10^−6^). (B) Regional association plots of genome-wide significant loci. Each dot represents an SNV and purple diamonds represent the index SNVs. Linkage disequilibrium with index SNV is indicated by r^2^. SNV=single nucleotide variant. *Did not reach p of less than 5 × 10^−6^ in the sensitivity analysis, and therefore was not assessed in further analyses.

We did a GWAS in our discovery cohort, including common variants and low-frequency variants (MAF_cohort_ ≥0·005), and identified four genome-wide significant loci associated with ADAD risk (figure 1A, table 2, appendix 2 p 11). The most significant locus was observed on chromosome 14 led by rs141931440 (p<0·0001, odds ratio [OR] 5·96 [95% CI 3·42–10·36]; MAFcohort 0·027; χ^2^ OR 4·94) and located near the *RHOJ* gene (figure 1B). The second significant locus was located on chromosome 1 led by rs74546314 (p<0·0001, OR 11·99 [5·39–26·64]; MAFcohort 0·005; χ^2^ OR 10·86), coding for a predicted pathogenic missense variant within *CNIH4* (Gly54Ser, combined annotation-dependent depletion score 25·9; figure 1B). The other two loci were identified on chromosome 5, led by the variant rs537168961 (p<0·0001, OR 9·56 [4·29–21·24]; MAFcohort 0·006; χ^2^ OR 9·30), located in an intergenic region near the *CCNG1* gene, and on chromosome 2, led by the variant rs78120946 (p<0·0001, OR 7·21 [3·56–14·57]; MAFcohort 0·009; χ^2^ OR 7·30), located in intronic regions of the *ALK* gene (figure 1B). The similarity between allele frequencies observed in the Genome Aggregation Database (gnomAD) database and those in control groups offers supportive evidence for the reliability and validity of our GWAS findings (table 2). No independent signals were detected after a stepwise conditional analysis on the four identified genome wide significant loci (appendix 2 p 12). We observed no effect of the *APOE* ε*4* status in regard to the significance of the detected associations (appendix 2 p 24). Sex-stratified analyses showed consistent associations for both sexes (appendix 2 p 25). We validated the *RHOJ* (p<0·0001, OR 6·11 [3·29–11·38]; MAF 0·026), *CCNG1* (p<0·0001, OR 9·78 [3·96–24·10]; MAF 0·006), and *CNIH4* (p<0·0001, OR 9·88 [3·83–25·46]; MAF=0·005) loci in sensitivity analyses including all 148 non-Hispanic White participants who were symptomatic carriers of ADAD mutations and 5813 control participants, including related participants (appendix 2 p 7). However, the signal at the *ALK* did not reach suggestive significance (p<5 × 10^−6^) in the sensitivity analyses and was therefore not further assessed. 18 participants carried the risk allele at the *RHOJ* locus, with 15 carrying a mutation in the *PSEN1* gene, one in the *PSEN2* gene, and two in the *APP* gene. Nine participants carried the risk allele at the *CNIH4* locus, with two carrying a *PSEN1* mutation and seven a *PSEN2* mutation. Finally, nine participants carried the risk allele at the *CCNG1* locus, with eight carrying a *PSEN1* mutation and one an *APP* mutation (appendix 2 p 26). We further stratified our analysis per gene and confirmed the cross-ADAD gene effect of the sentinel variants (appendix 2 pp 7, 27). Furthermore, including *APOE* ε*4* as an interaction term with the sentinel variants showed no significant effect (appendix 2 p 28). Overall, these variants increased Alzheimer’s disease risk in ADAD mutation carriers regardless of the ADAD gene affected. Finally, in the replication analysis in 34 unrelated ADAD case participants and 8249 control participants from ADSP R5, rs141931440 (*RHOJ*) showed nominal significance (p=0·036, OR 3·01 [1·07–8·46]; MAF 0·019). In the meta-analysis of the discovery and ADSP R5 replication datasets, *RHOJ* (p<0·0001, OR 5·11 [3·13–8·34]) and *CNIH4* (p<0·0001, OR 10·16 [4·73–21·84]) sentinel variants reached genome-wide significance, and the *CCNG1* sentinel variant was nearly genome-wide significant (p<0·0001, OR 7·47 [3·61–15·41]; appendix 2 p 29). Furthermore, the sentinel variants were not associated with sporadic Alzheimer’s disease (appendix 2 p 30). In the burden tests of the four implicated genes, only the *CNIH4* gene showed a significant association (p<0·0001), driven primarily by the sentinel variant at this locus.

To functionally characterise the Alzheimer’s disease sentinel variants, we leveraged protein QTL data generated for 7388 proteins using plasma from 2338 Knight-ADRC participants. rs537168961 (*CCNG1*) was identified as a significant protein QTL for TDP-43 (p<0·0001, β=0·089; appendix 2 p 29, figure 2A), and rs141931440 (*RHOJ*) was associated with myeloid cell surface antigen CD33 levels (p<0·0001, β=–0·13; appendix 2 pp 14–15, 31). We validated the effect of the rs537168961 allele on plasma TDP-43 levels using NULISA protein-level data from the Knight-ADRC cohort (2123 participants) and revealed that the rs537168961 allele was associated with increased TDP-43 levels (p=0·020).

We assessed whether the sentinel variants were located in cis-regulatory regions linked to neurodegenerative diseases by integrating Vertebrate Regulatory Module Detector predicted cis-regulatory modules, a multiple expression QTL dataset, and experimentally defined and putative regulatory sequences. Only one sentinel variant, rs141931440, was located within a human predicted cis-regulatory module with two lines of evidence supporting its regulatory functionality: a DNase hypersensitive peak and an H3K27ac peak (appendix 2 p 16). This cis-regulatory module was located upstream of the *RHOJ* gene. rs141931440 is indeed an expression QTL in the brain frontal cortex with the T allele associated with decreased *RHOJ* expression (p=0·0073, GTEx database). We detected the presence in the *RHOJ* region of independent SNVs altering *RHOJ* RNA expression (appendix 2 pp 16–17). *RHOJ* expression was consistently downregulated in multiple astrocyte subpopulations across three neurodegenerative diseases (Alzheimer’s disease, Huntington’s disease, and Nasu–Hakola disease), suggesting astrocyte-specific *RHOJ* function in neurodegeneration (appendix 2 pp 7, 18).

**Figure 2:**
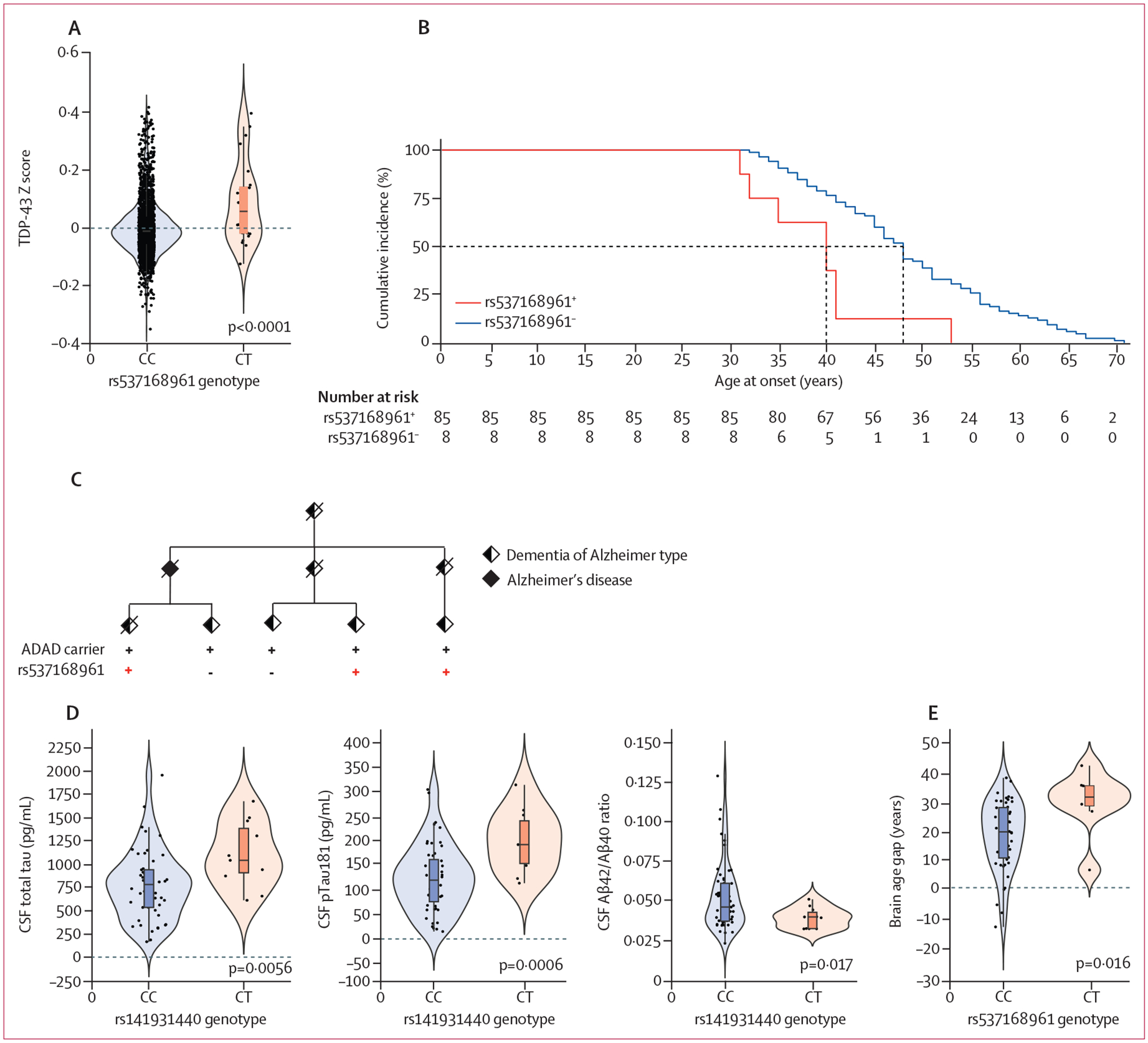
Statistical and functional analysis of sentinel variants (A) TDP-43 plasma protein level measurement for rs537168961 (*CCNG1*) CT and CC among Knight-ADRC participants. (B) Cumulative incidence among rs537168961 (*CCNG1*) CT and CC. All participants are ADAD mutation carriers. (C) Segregation analysis. In this pedigree, diamonds represent individuals with sex masked for privacy. Deceased individuals are marked with a crossed bar. The age at onset is shown for each individual. A “+” sign indicates carriers of the ADAD mutation, and a red “+” sign indicates CT. The mean age at onset of participants carring both the ADAD mutation and the risk allele is 39 years, and that of participants carrying the ADAD mutation but not the risk allele is 47 years. (D) CSF Alzheimer’s disease biomarker measurements for CSF total Tau, pTau181, and amyloid β plaque burden for rs141931440 (*RHOJ*) CT and CC among participants carrying an ADAD mutation. Dots represent participants, with orange for those carrying the rs141931440 risk allele (CT) and blue for those who are homozygous for the rs141931440 reference allele (CC). (E) Neuroimaging brain age gap analysis for rs537168961 (*CCNG1*) CT and CC among participants carrying the ADAD mutation. Dots represent participants, with orange for those carrying the rs537168961 risk allele (T) and blue for those who are homozygous for the rs537168961 reference. p represents the linear regression model significance in (A), (D), and (E), and the log-rank test statistical significance in (B). ADAD=autosomal dominant Alzheimer’s disease. CC=non-carriers of the indicated risk allele. CT=carriers of the indicated risk allele.

We then assessed the effect of our sentinel variants on AAO. rs537168961 (*CCNG1*) was associated with a significant decrease in the age at dementia onset in participants carrying an ADAD mutation (p=0·0068, β=–10·15 [95% CI −17·31 to −2·77]; table 3, appendix 2 p 19). *CNIH4* (p=0·49, β=2·52 [–4·53 to 9·58]) and *RHOJ* (p=0·47, β=–2·09 [–7·74 to 3·55]) loci did not show significant association with AAO. Adding *APOE* ε*4* status as a covariate did not significantly change the results, with the *CCNG1* locus remaining significant (p=0·013, β=–9·40). Additionally, stratifying by sex did not substantially change our findings (appendix 2 p 32). None of the carriers of the risk alleles carried an additional risk allele from another locus; therefore, the synergic effect of these variants could not be assessed. Kaplan–Meier survival analysis resulted in a significant association exclusively with the rs537168961 (*CCNG1*) risk allele, showing that carriers of the risk allele had a median AAO of 40 years compared with 48 years among non-carriers (p=0·0009; figure 2B, table 3). Moreover, Cox proportional hazards regression analysis identified a significant association between rs537168961 (*CCNG1*) and earlier Alzheimer’s disease onset, with a hazard ratio (HR) of 5·72 (p=0·0065; table 3). *CNIH4* (p=0·84, HR 1·08 [95% CI 0·51–2·24]) and *RHOJ* (p=0·58, HR 1·20 [0·63–2·27]) sentinel variants showed positive effect directions, suggesting that they might be associated with an increased risk of earlier AAO. In a large family with the *PSEN1* pathogenic mutation and several carriers of rs537168961, the average AAO for individuals carrying both the *PSEN1* mutation and the rs537168961 (*CCNG1*) risk allele variant was 38·7 years (three participants; figure 2C), whereas it was 47 years for participants carrying the *PSEN1* mutation but not the rs537168961 (*CCNG1*) risk allele (two participants). We assessed this variant in the ADSP R4 dataset and did not observe an association with AAO in sporadic cases.

Participants with ADAD in the DIAN cohort carrying the risk allele in the *RHOJ* locus (rs141931440) had significantly elevated levels of total Tau (p=0·0056, β=358·37) and pTau181 (p=0·0006, β=81·28), and a significantly reduced Aβ42/Aβ40 ratio (p=0·016, β=−0·11), compared with ADAD participants without the risk allele (figure 2D, appendix 2 p 30). Overall, the rs141931440 risk allele was associated with Alzheimer’s disease-related pathology and more advanced stages of Alzheimer’s disease, whereas the other two sentinel variants were not.

Finally, for the 64 DIAN participants, no significant association was identified between the sentinel variants and amyloid imaging, cerebral metabolic rate of glucose, hippocampal volume, and cortical thickness (appendix 2 p 34). We also assessed the effect of the sentinel variants with the brain age gap. We detected that the rs537168961 (*CCNG1*) locus was significantly associated with a higher brain age gap (p=0·016, β=11·34; figure 2E).

## Discussion

To our knowledge, we carried out the largest GWAS on individuals symptomatic for Alzheimer’s disease carrying *PSEN1, PSEN2*, and *APP* mutations to date, and discovered three novel loci that further increase the risk of Alzheimer’s disease in ADAD. Our study highlighted two loci located in non-coding regions near the *CCNG1* and *RHOJ* genes, and a third locus led by a non-synonymous variant (Gly54Ser) in the *CNIH4* gene. Our results show that, even in the presence of almost fully penetrant ADAD mutations, additional strong genetic factors play an important role in disease progression and aetiology.

Altogether, these new risk alleles are present in 27% of individuals carrying ADAD mutations and are associated with increased disease risk across mutations and genes. This finding is different from previously reported genetic modifiers in ADAD. In fact, the *APOE* Christchurch and *RELN* variants, which were reported in large families with the same mutation, are yet to be replicated in independent families due to their low MAF.^6,7^ Whereas the *APOE* Christchurch and *RELN* variants were protective, individuals carrying an ADAD mutation and one of these newly identified common risk alleles face an even higher risk of developing Alzheimer’s disease compared with non-risk allele carriers. This finding suggests a potential multistep genetic process, similar to those reported for amyotrophic lateral sclerosis and sporadic early onset Alzheimer’s disease.^20,21^

The *CCNG1* locus was also associated with reduced onset age by an average of 10 years and larger brain age gap, highlighting its potential impact on both risk and presentation of Alzheimer’s disease. This AAO effect is substantially stronger than the effect size of 1–6 years from the *SNX25* variants identified in the Caribbean Hispanic Gly206Ala *PSEN1* mutation,^11^ or even in sporadic Alzheimer’s disease cases, where each *APOE* ε*4* allele significantly reduces the AAO by 2·45 years.^22^ In our study, we observed a marginal effect of the *APOE* ε*4* allele suggesting a potential divergent role of *APOE* in ADAD compared with sporadic Alzheimer’s disease. *CCNG1* encodes cyclin G1, which is crucial in cell cycle regulation due to its interaction with various cyclindependent kinases and phosphatases to control cell cycle progression and maintain genomic stability. In our study, the *CCNG1* risk allele was positively correlated with plasma TDP-43 levels. Dysregulated cyclindependent kinase activity leads to cell death and accumulation of TDP-43.^23^ In Alzheimer’s disease mouse models, TDP-43 depletion worsens neurodegeneration and alters amyloid β dynamics, whereas overexpression in models with familial Alzheimer’s disease mutations promotes changes in amyloid plaques and increases tau aggregation. In 2025, Wang and colleagues^24^ reported that increased plasma TDP-43 is associated with advanced limbic-predominant age-related TDP-43 encephalopathy neuropathological change, suggesting that carriers of the *CCNG1* risk allele—showing higher plasma TDP-43 levels—could have increased TDP-43 pathology in their brain. However, the effect of the *CCNG1* risk allele on TDP-43 pathology and the role of TDP-43 pathology on AAO needs to be further assessed. Understanding the biological mechanisms between CCNG1 and TDP-43 could guide the development of future therapeutic strategies designed to modulate TDP-43 accumulation, with the potential to slow or prevent disease progression.

On the other hand, the rs141931440 (*RHOJ*) risk allele was associated with increased CSF total tau and pTau181 levels, along with a reduced Aβ42/Aβ40 ratio. These findings suggest that the presence of the risk allele exacerbates amyloid β pathology. The *RHOJ* gene encodes for Ras Homolog Family member J, which is involved in focal adhesion of endothelial cells. *RHOJ* is regulated by vascular endothelial growth factor and together they influence angiogenesis, which might play a role in polygenic Alzheimer’s disease resilience.^25,26^ Furthermore, the *RHOJ* gene is highly expressed in astrocytes and downregulated in Alzheimer’s disease (appendix 2 pp 7, 18). Astrocytes are a major player for amyloid β clearance, but in Alzheimer’s disease, reactive astrocytes can also contribute to amyloid β production due to their altered state caused by neuroinflammation.^27,28^ Furthermore, astrocytic activation has been reported to coincide with early fibrillar amyloid β plaque deposition.^27,28^ The rs141931440 variant might affect RHOJ astrocytic function and increase Alzheimer’s disease pathology. Additionally, we found that the *RHOJ* risk allele is a plasma protein QTL for the *CD33* gene, which is extensively studied in connection with Alzheimer’s disease due to its role in microglial activity and involvement in various disease pathways, including immune response regulation and phagocytosis.^29^ Further research is essential to disentangle these molecular mechanisms.

The signal at the *CNIH4* locus, driven by the rs74546314 missense variant, encodes for the cornichon family member 4, which is involved in G protein-coupled receptor trafficking. The *CNIH4* gene has been implicated in cancer progression by reducing irondependent programmed cell death. This process attenuates amyloid β plaque load and contributes to neurodegeneration in Alzheimer’s disease,^30,31^ suggesting that *CNIH4* could contribute to Alzheimer’s disease through ferroptosis, but additional studies are necessary to formally implicate *CNIH4* in Alzheimer’s disease aetiology.

This study has several limitations that should be considered. First, despite having the largest cohort to date, the rarity of ADAD results in a relatively small sample size, which might potentially reduce the statistical power of our analysis. However, our assessment indicates that we had between 48% and 95% power to detect the significant loci identified. Although we provided additional biological evidence suggesting that the *RHOJ* and *CCNG1* loci are associated with disease aetiology, the sample size might have influenced our results and associations at the *CNIH4* and *ALK* loci should be interpreted with caution, as they might be affected by the winner’s curse effect. Second, our analysis is based on participants of European descent, which restricts the generalisability of our findings to other populations. Future studies incorporating multiancestry participants and larger cohorts are essential to enhance the applicability of our results across diverse populations. Overall, such studies would not only help validate our findings but also enable the identification of additional genetic modifiers.

In summary, we discovered three new Alzheimer’s disease risk loci in *CCNG1*, *RHOJ*, and *CNIH4*. The *CCNG1* variant is associated with earlier onset, potentially through the acceleration of pathogenic processes such as TDP-43 aggregation, providing potential insight into the biology driving early neurodegeneration. The *RHOJ* variant is associated with CSF Alzheimer’s disease biomarkers, highlighting its importance in the disease process, whereas *CNIH4* suggests the potential role of ferroptosis in Alzheimer’s disease risk. These genetic variants could help family genetic counselling and future clinical trial designs. This study deepens our understanding of the genetic factors influencing Alzheimer’s disease progression and opens new avenues for research and therapeutic development.

## Supplementary Material

Supplementary Appendix

DIAN Author List

## Figures and Tables

**Table 1:** Demographic information for the discovery cohort

	Control participants (n=5050)	Total case participants (n=101)	Case participants with an *APP* mutation (n=15)	Case participants with a *PSEN1* mutation (n=79)	Case participants with a *PSEN2* mutation (n=7)
Sex					
Female	3019 (60%)	46 (46%)	9 (60%)	32 (41%)	5 (71%)
Male	2031 (40%)	55 (54%)	6 (40%)	47 (59%)	2 (29%)
Age, years	80 (73–86)	47 (40–55)	48 (43–55)	46 (38–54)	50 (46–55)
Participants with 1–2 copies of *APOE* ε4	1411 (28%)	34 (34%)	8 (53%)	20 (25%)	6 (86%)

Data are n, n (%), or median (IQR). The discovery cohort comprised non-Hispanic White participants included in case–control genome-wide association study analysis. Age is given as median age at the last healthy visit for control participants and median age at onset for case participants.

**Table 2:** Genome-wide significant loci

	rsID	SNV	p	OR (95% CI)	MAF cases/controls	MAF NFE
*CNIH4*	rs74546314	1:224365900:G:A	1·05 × 10^−9^	11·99 (5·39–26·64)	0.044/0·004	0·003
*ALK*	rs78120946	2:29324788:T:A	3·78 × 10^−8^	7·21 (3·56–14·57)	0.054/0·008	0·011
*CCNG1*	rs537168961	5:162973378:C:T	3·05 × 10^−8^	9·56 (4·29–21·24)	0.045/0·005	0·008
*RHOJ*	rs141931440	14:63196126:C:T	2·77 × 10^−10^	5·96 (3·42–10·36)	0.089/0·021	0·018

Genome-wide significant loci (p<5·0 × 10^−8^) identified in case–control genome-wide association study analysis. rsID=Single Nucleotide Polymorphism Database reported variant identification number. SNV=single nucleotide variant (chromosome [GRCh38], base-pair position, reference allele, alternative allele). OR=odds ratio. MAF=minor allele frequency. NFE=Genome Aggregation Database (gnomAD) non-Finnish European.

**Table 3:** Effect of sentinel variants on age at onset

	rsID	Linear model		Linear model (*APOE* ε4 adjusted)		Kaplan-Meier	Cox regression	
		p	β (95% CI)	p	β (95% CI)	p (log-rank)	p	Hazard ratio (95% CI)
*CNIH4*	rs74546314	0·49	2·52 (−4·53 to 9·58)	0·68	1·52 (−5·79 to 8·48)	0·90	0·84	1·08 (0·51–2·24)
*CCNG1*	rs537168961	0·0068	−10·15 (−17·31 to −2·77)	0·013	−9·40 (−16·67 to −2·13)	0·0009	0·0065	5·72 (1·62–20·08)
*RHOJ*	rs141931440	0·47	−2·09 (−7·74 to 3·55)	0·56	−1·66 (−7·35 to 4·02)	0·83	0·58	1·20 (0·63–2·27)

Results from the age at onset (linear model), adjusted *APOE* ε4 (linear model), Kaplan–Meier survival, and Cox proportional hazard risk ratio analyses of the top risk variants. rsID=Single Nucleotide Polymorphism Database reported variant identification number.

## Data Availability

Summary statistics from the GWAS in this study are available at GWAS Catalog (GCST90727422).

## Research in context

### Evidence before this study

We searched PubMed on March 1, 2023, for publications published from database inception to March 1, 2023, using the terms “APP” OR “PSEN1” OR “PSEN2” AND “onset age variability”, without language restrictions. We identified 79 studies, which reported large variability in age at onset (AAO) of autosomal dominant Alzheimer’s disease (ADAD) among individuals carrying *PSEN1*, *PSEN2*, or *APP* mutations, suggesting that genetic modifiers might be in part responsible for the phenotypic presentation. We also searched PubMed using various terms including “genome-wide association study”, “Alzheimer’s disease”, and “genetics”, and curated relevant publications aimed at genetic modifier identification, yielding six studies. These studies on large families identified potential genetic modifiers, such as *APOE* Christchurch and *RELN*, that influence AAO of ADAD. Other studies leveraged founder mutations and identified potential genetic modifiers, which influence key pathological processes such as amyloid deposition, neuronal resilience, and synaptic function. However, replication is still insufficient, the effect of these potential genetic modifiers across ADAD mutations remains to be assessed, and additional modifiers remain to be identified.

### Added value of this study

In our study, to the best of our knowledge, we gathered the largest ADAD cohort in the world, increasing not only the size of ADAD studies for genetic modifiers but also the diversity of mutations compared with previously reported studies. Through a genome-wide association study, we identified three new genetic loci acting as risk modifiers of the three ADAD genes. Among these loci, one variant is located in the coding region of the *CNIH4* gene. The sentinel variant at the *CCNG1* locus was associated with earlier AAO, potentially through TDP-43 pathology. The sentinel variant at the *RHOJ* locus was associated with CSF Alzheimer’s disease biomarkers, highlighting its role in the progression of the disease.

### Implications of all the available evidence

The identification of these genetic variants offers promising new pathways for developing biomarkers and therapeutic strategies for individuals with ADAD. Additionally, the variants could significantly improve genetic counselling for affected families and aid in the planning of future clinical trials. This research enhances our understanding of the genetic factors that drive Alzheimer’s disease progression and paves the way for further study and therapeutic innovation.
