# Supplementary material for: Identification of genetic modifiers of autosomal dominant Alzheimer’s disease: a genome-wide association study
Source: Lancet Neurol. Author manuscript; Available in PMC 2026 Jul 7. (PMC13340468; doi:10.1016/S1474-4422(26)00123-7)
Supplement: Supplementary Appendix [file NIHMS2189320-supplement-Supplementary_Appendix.pdf]

THE LANCET
Neurology

Supplementary appendix 2

This appendix formed part of the original submission and has been peer reviewed. We post it as supplied by the authors.

Supplement to: Patel M, Feng W, Mckay NS, et al. Identification of genetic modifiers of autosomal dominant Alzheimer's disease: a genome-wide association study. *Lancet Neurol* 2026; **25:** 581–90.

Table of Contents

1. SUPPLEMENTARY METHODS 3

Sample processing, genotyping and quality assessment 3

Selection of case (ADAD carriers) and control participants 3

Identity by descent and population stratification analysis 4

Genome-wide association analysis 4

Protein quantitative trait loci analysis 4

Identification of cis-regulatory effect and chromatin accessibility 5

Age at onset analysis 6

Segregation analyses 6

Cerebrospinal fluid biomarker analysis 6

Neuroimaging analyses 6

2. SUPPLEMENTARY RESULTS 7

Genome-wide association analysis 7

Cis-regulatory effect of the sentinel variants 7

3. SUPPLEMENTARY FIGURES 8

Figure S1: Identical by descent (IBD) analysis of the participants including in the unrelated Non-Hispanic White study. 8

Figure S2: Principal component analysis of case-control participants with 1000 genome as a reference population. 9

Figure S3: Schematic representation of the sample selection for the ADAD analysis 10

Figure S4: QQ-Plot of genome-wide significant analysis. 11

Figure S5: Locuszoom plot of step-wise conditional analysis of the identified sentinel variant of each locus. 12

Figure S6: Minor allele frequency of sentinel variants in cases and controls. 13

Figure S7: CD33 protein level in plasma among knight-ADRC participants. 14

Figure S8: Correlation plot of ADAD GWAS and pQTL datasets 15

Figure S9: rs141931440 (*RHOJ* locus) is located within a predicted cis-regulatory module. 16

Figure S10: Correlation Plot between ADAD GWAS (rs141931440) and eQTL GTEx brain frontal cortex (BA9) dataset. 17

Figure S11: Differential expression of the *RHOJ* gene in astrocytes under various disease conditions. 18

Figure S12: Age at onset distribution of carriers and non-carriers of the *CCNG1* sentinel (rs537168961) risk allele. 19

4. SUPPLEMENTARY TABLES 20
Table S1: List of mutations in *APP*, *PSEN2* and *PSEN1* genes. 20
Table S2: Demographic information 23
Table S3: Genome-wide significant loci with and without adjusting for *APOE ε4* 24
Table S4: Sex stratified analysis (case/control association study) for the genome-wide significant sentinel variants. 25
Table S5: Carriers of sentinel variants and their ADAD mutations. 26
Table S6: Association study stratified by ADAD genes for sentinel variants. 27
Table S7: Association analysis stratified by ADAD gene with *APOE ε4* interaction term. 28
Table S8: Replication and meta-analysis using ADSP R5 datasets. 29
Table S9: Case/Control GWAS analysis of sporadic AD using ADSP datasets. 30
Table S10: Plasma pQTL analysis of risk loci 31
Table S11: Sex stratified analysis on age at onset (AAO) of genome-wide significant loci. 32
Table S12: Linear regression analysis with Lumipulse CSF AD biomarkers: CSF tTAU, CSF pTau181, and CSF Aβ42/40. 33
Table S13: Demographics of participants with and without neuroimaging 34
5. References: 35
6. Acknowledgements: 37

1. SUPPLEMENTARY METHODS

Sample processing, genotyping and quality assessment

Whole-genome sequencing and analysis of Knight-ADRC and DIAN cohort

Whole-genome sequencing was performed on 2,464 extracted DNA samples from Knight-ADRC and DIAN cohorts using the Illumina TruSeq DNA Sample Preparation Kit on Illumina HiSeq2000/Novaseq6000 platforms. The sequencing targeted an average read depth of 30x and was conducted at various sequencing centers including McDonnell Genome Institute (MGI), Uniformed Services University of the Health Sciences (USUHS), Broad Institute, Genentech or Illumina sequencing center. The resulting high-quality reads were aligned with the human reference genome, GRCh38, using Burrows-Wheeler Aligner (v0.7.17).[1] Variant calling was performed according to GATK's (v4.1.1) best practices, resulting in unified VCF files and stringent quality control process (https://software.broadinstitute.org/gatk/best-practices/).[2] We used the GenotypeGVCFs tool in GATK to merge the intermediate genomic VCF (gVCF) files into a unified VCF file containing the Knight-ADRC and DIAN datasets (n=2,464). We used BCFTOOLS to decompose multiallelic variants into bi-allelic variants.[3] We applied GATK variant quality score recalibration (VQSR) to WGS SNVs and insertion/deletions (indels). Only SNVs and indels with a confidence threshold above 99·9% were included in the analysis. Variants located in regions of low complexity were excluded, and variants with a depth (DP) exceeding the average DP plus 5 standard deviations of the WGS dataset were also excluded. We removed non-polymorphic variants and variants that did not fall within the expected allelic balance ratio for heterozygous calls (ABHet=0·25 to 0·75). We then applied several hard filtering criteria to SNVs and indels: (1) quality depth (QD) ≥ 7 for indels and QD ≥ 2 for SNVs, (2) mapping quality (MQ) ≥ 40, (3) FisherStrand (FS) balance ≥ 200 for indels and FS ≥ 60 for SNVs, (4) strand odds ratio (SOR) ≥ 10 for indels and SOR ≥ 3 for SNVs, (5) inbreeding coefficient (IC) ≥ −0·8 for indels, and (6) ReadPosRankSum (RPRS) test to measure the relative position of reference and alternative alleles within the reads: RPRS ≥ −20 for indels and RPRS ≥ −8 for SNVs. These filters resulted in 41,514,968 high quality genetic variants.

Whole-genome sequencing and analysis of ADSP R4

We further included the Alzheimer Disease Sequencing Project quality-controlled whole genome dataset (ADSP R4, NG00067.v10, n=16,549 (Controls and ADAD mutation carriers)).[4] Briefly, ADSP R4 genotype calling was performed for each variant across all participants followed by joint genotyping on all SNVs and short indels using GATK’s best practices. Subsequently, multi-allelic variants were converted to bi-allelic variants on autosomes. Variants with a GATK “FILTER” status of PASS or is within a tranche of ≥ 99·8%, with DP > 10, GQ > 20, call rate of ≥ 80%, and supported by fewer than 500 reads were retained.

Merged whole-genome datasets

The ADSP R4 filtered individual dataset was merged with the Knight-ADRC and DIAN cohorts based on the genetic variants present across all cohorts, which resulted in a total of 19,013 participants with 23,696,524 high quality variants. Additional variant quality control for the Knight-ADRC/DIAN and ADSP cohorts was performed using Plink (v2.0)[5]. We assessed allele balance and applied Hardy-Weinberg equilibrium filtering using a p-value threshold of 1×10^{-30}. We applied stringent thresholds for genotype missingness (98%) and individual missingness rate of 98% to filter out variants and participants with high genotyping missingness.

Selection of case (ADAD carriers) and control participants

After sequencing, variant calling, and quality controls, rare variants within the *PSEN1*, *PSEN2* and *APP* genes were retrieved and annotated as being pathogenic or likely pathogenic based on ClinVar (https://www.ncbi.nlm.nih.gov/clinvar/), ALZFORUM (https://www.alzforum.org/mutations), and DIAN mutation databases (table S1 in appendix). Case participants were defined as symptomatic and carrying pathogenic or likely pathogenic mutations in the ADAD genes (Clinical Dementia Rating (CDR > 0·5)), while control participants were free of neurodegenerative diseases. Participants with mild cognitive impairment (CDR=0·5, n=2), presymptomatic carriers under 60 years (n=154), and those without age data (n=14) were excluded, resulting in a dataset of 248 symptomatic participants with an ADAD mutations and 18,595 controls.

Identity by descent and population stratification analysis

To select unrelated participants, we performed identity by descent (IBD) calculations using KING Robust to remove duplicate individuals (n=754) with PI-HAT score greater than 0·85 and we selected only one participant per family using the recommended kinship coefficient threshold of 0.0442 in KING matrix[6] and prioritized participants with the earliest age at onset (AAO) for cases and latest age at healthy assessment for controls. In total, we identified 1,350 related individuals (figure S1 in appendix). To select individuals of non-Hispanic Whites (NHW), we performed principal component analysis (PCA) using common variants (MAF>0·02) and population data from the 1000 Genomes Project (https://www.internationalgenome.org/) as a reference panel (figure S2 in appendix). We identified 5,151 unrelated NHW ancestry participants. In the control group, 60·17% of the participants were female, while in the case group, 45·54% of the participants were female.

Genome-wide association analysis

We assessed the association between SNVs ($MAF_{cohort} \geq 0{\cdot}005$) and disease status by fitting a logistic Firth regression model in Plink (v2.0) and adjusting for sex, cohort, and the first 10 principal components (PCs). To create Manhattan and quantile-quantile plots, we used the QQman (v 0.1.9) package in R. We used the Locuszoom standalone software from the University of Michigan to generate the Locuszoom plot [https://genome.sph.umich.edu/wiki/LocusZoom_Standalone]. To assess potential independent signals at these loci, we performed a conditional logistic regression analysis for each genome-wide significant sentinel variant, adjusting for the same covariates used in single variant analysis. Additionally, in order to leverage the related individuals, we generated a sparse genetic relationship matrix (SPARS GRM) to account for sample relatedness and applied a saddlepoint approximation test controlling for the unbalanced case/control composition of our cohort. We conducted a sensitivity analysis on the top identified SNVs ($p<5{\cdot}0\times10^{-8}$) using SAIGE on unbalanced case-controls, including related cases (table S2 in appendix). Both sensitivity analyses were adjusted for sex, cohort, and the first 10 PCs. Additionally, to determine if the sentinel variants were associated with sporadic AD, we performed a case/control analysis using the ADSP R4 NHW dataset (N cases: 6,547; N controls: 4,739). We included sex, PC1-PC10, and sequencing center as covariates. We performed per-gene stratified analysis for each sentinel variant to assess the effect of each variant per ADAD gene. The models were also adjusted for covariates such as sex, PC1-PC10, and cohort.

In order to perform replication analysis, we utilized the ADSP R5 datasets that consists in 15,085 cases and 30,239 controls. We utilized the preview project VCFs from the NIAGADS 2025-11 release. The variants were jointly called using GLNexus. We set genotypes with DP < 10 or GQ < 20 to missing, removed variants with GLNexus QUAL scores < 100, removed variants in low complexity regions, removed monomorphic variants, removed variants with mean read depth > 500, removed variants with allelic balance ratio > 0·75 or < 0·25, split multiallelics, and removed variants with genotyping rate < 95%. We conducted a PCA to identify participants.

We then subset the dataset to NHW cases with ADAD mutations and controls (n=151, n=22,832, respectively). To remove duplicate and related individuals as compared to the discovery phase, we merged the discovery datasets with the replication datasets. We then employed the KING software with identical parameters used in the discovery datasets. Through this process, we identified 34 unrelated cases and 8,249 controls NHW participants non-overlapping and unrelated with the discovery set. We then performed case-control analysis adjusted for sex and the first ten principal components (PC1-PC10) to account for potential confounding factors.

Protein quantitative trait loci analysis

Effect of sentinel variants on plasma protein levels was assessed using previously generated pQTL data from 2,338 Knight-ADRC and DIAN participants.[7] This dataset included sporadic AD patients, ADAD, healthy controls, frontotemporal dementia patients, and individuals with an unclassified neurodegenerative disease. Briefly, plasma samples were collected in the morning without fasting. All samples were centrifuged immediately following the same protocols and stored at −80°C. To minimize batch effects, the samples were sent randomly distributed across different plates. Protein levels were measured using the SomaLogic aptamer-based SomaScan platform (Somascan 7k), which employs a multiplexed single-stranded DNA aptamer assay for protein quantification. The resulting data includes quantitative levels of approximately 7,000 aptamers, measured in relative fluorescence units (RFU). Initial data normalization was performed by SomaLogic, which utilized hybridization controls for intra-plate normalization and median signals to address inter-plate variability. SomaLogic also implemented an additional normalization step, where data is normalized against an external reference to control for biological variation.[8] Quality control at both the aptamer and individual levels was subsequently carried out to detect and exclude outlier analytes and samples, using a in-house developed pipeline.[8,9] These datasets were utilized in the pQTL analysis. We queried the sentinel variants for their

association with plasma protein levels[10,11] using the WASHU Online Neurodegenerative Trait Integrative Multi-Omics Explorer (ONTIME) (https://ontime.wustl.edu/).

Additionally, the effect of the sentinel variants on transactive response DNA-binding protein 43 (TDP-43; t22718) plasma protein levels was assessed using previously generated plasma Alamar protein level data from 2,123 Knight-ADRC participants. Samples were stored at -80°C until ready for use. Before the assay, samples were thawed and centrifuged at 10,000g for 10 min. 10 µL supernatant from the sample was plated in 96-well plates and assayed with the NULISAseq CNS Panel which targets 123 specific proteins. Data normalization comprises several crucial steps to ensure precise statistical analysis. Initially, the normalization process begins with internal control adjustment, where the target counts for each sample are divided by the internal control counts of that sample. Following this, Inter-Plate Control (IPC) normalization is performed, which involves calculating the medians of target-specific IPC replicates on each plate. These median values are then used to further normalize the IC-adjusted counts. The normalized counts are rescaled by multiplying by a factor of 10^4. Finally, to achieve a more normal distribution, a log2 transformation is applied; before this transformation, all values are incremented by one. The resulting transformed values are referred to as NULISA Protein Quantification (NPQ) units. Protein concentrations, reported in NULISA Protein Quantification (NPQ) units, were normalized for intraplate and intensity variability and then log2-transformed to approximate a normal distribution. No additional transformations were applied. Data points that fell outside 1.5 times the IQR from either the first (Q1) or third (Q3) quartile were identified as outliers and replaced with NAs. The call rates, indicating the proportion of successful measurements, were computed for each analyte and sample. A two-step filtering method based on call rates was employed, starting with a threshold of 65% followed by 85%, to exclude lower-quality data. Call rates were recalculated after applying the 65% threshold (high rate of missingness) and before applying the 85% threshold. This method aimed to retain borderline analytes and samples. After QC, the final dataset included 123 analytes. Although the limit of detection (LOD) and coefficient of variation (CV) were evaluated for each analyte, these parameters were not used as filters. Following QC, the default log2-transformed NPQ values were reverted to linear NPQ values to allow end users to implement their preferred normalization. For this study, the linear NPQ values were log10 transformed. We performed the linear regression analysis between sentinel variants and of transactive response DNA-binding protein 43 (TDP-43; t22718) plasma Alamar protein levels, adjusting for age and gender as a covariates.

Identification of cis-regulatory effect and chromatin accessibility

We then assessed the effect of the sentinel variants on gene regulation. We gathered Cis regulatory modules (CRM) from two sources: 1) manually curated 97 experimentally defined CRMs for mouse and 60 modules for human through an extensive literature search using keywords such as "cis-regulatory modules", "Single-cell ATAC-seq" and "expression quantitative trait loci" in Pubmed. 2) We downloaded 998 experimentally defined enhancers that exhibited enhancer activity from VISTA Enhancer Browser for the human genome.[12] Human predicted enhancers were obtained from Enhancer Atlas 2.0 (http://www.enhanceratlas.org/data/download/species_enh_csv.tar.gz). From the Cistrome database, chromatin accessibility (ATAC-seq and DNAse-seq) peaks and ChIP-seq histone marks (H3K27ac, H3K4me1, and H3K4me3) peaks were downloaded (http://cistrome.org/db/batchdata) for human genomes. ENCODE human cCREs are available at the web-based server "Search Candidate cis-Regulatory Elements by ENCODE V3" (SCREEN; http://screen.encodeproject.org).[13] Human scATAC-seq can be accessed at their portal http://catlas.org/humanenhancer/#!/.[14] Each dataset included peaks from hundreds of different tissues and cell types. Files from the same type of data were merged using bedtools merge to eliminate overlapping elements. Modules bigger than 2·5 kb after merge were eliminated before comparison. We define an experimentally defined module as being correctly predicted at two different cutoffs: 1) experimentally defined modules overlap with predicted CRMs by 50% of the length of the shorter one; 2) they overlap by 1 bp. The odds ratio, confidence interval, and p-value (P) (chi-square independent test) were calculated comparing CRMsub and CTRL of each dataset. Peak-detection sensitivity and odds ratio were calculated using the R package fmsb. The difference was considered significant with an OR>1 and P<0.05. Data was visualized using https://epigenomegateway.wustl.edu/browser/?sessionFile=https://wangftp.wustl.edu/~dli/gzhao/CRMs-202207/hg38-s.json. Additionally, we investigated the rs141931440 risk allele effect on *RHOJ* using the expression quantitative trait loci (eQTL) data from the brain frontal cortex (BA9) available through the GTEx portal eQTLs datasets.[15]

We collected recently published eQTL datasets with thousands of significant neurodegenerative diseases-associated SNVs from four resources. 1) The Religious Orders Study and Memory and Aging Project (ROSMAP) Study is a longitudinal clinical-pathologic cohort study of aging and AD;[16] 2) The Brain xQTLServer includes data from

transcriptomic and epigenomic data for AD, Schizophrenia, and Bipolar Disorder;[17] 3) The Online Neurodegenerative Trait Integrative Multi-Omics Explorer (ONTIME) includes various quantitative neurodegenerative disease endophenotypes such as AD, Parkinson disease (PD), Frontotemporal Dementia, Amyotrophic Lateral Sclerosis, Dementia with Lewy Bodies, and Multiple Sclerosis;[18] 4) MetaBrain, a large scale eQTL meta-analysis of previously published human brain eQTL datasets.[19] MetaBrain includes AD data from different brain regions and from donors with different ancestry such as African, European, and East Asian. Only eQTLs that met the statistical significance threshold for the original study were included in our analysis.

Age at onset analysis

Age at onset (AAO) analyses were performed on symptomatic ADAD mutation carriers. We selected the sentinel variants and performed an AAO analysis using a linear regression model in Plink (v 2.0), adjusting for sex, PC1-PC10, and cohort. We applied a Bonferroni correction for the tested 3 SNVs ($0{\cdot}05/3=1{\cdot}7\times10^{-02}$). To follow up on AAO analysis, Kaplan-Meier survival analysis was performed to estimate the probability of disease onset times between carriers and non-carriers using the survival (v3.8-3) and survminer (v 0.5.0) packages in R.[20] We used the ggplot2 (version 3.5.1) R package to generate a Kaplan-Meier survival plot. The log-rank test was used to assess the statistical significance of differences in AAO between the groups. Moreover, we performed a Cox proportional hazard ratio analysis and compared the effect of the rare allele on AAO. In this analysis we included related participants and corrected for family identity. We applied a Bonferroni correction of $0{\cdot}05/3=1{\cdot}7\times10^{-02}$.

Segregation analyses

We conducted segregation analyses for each sentinel variants and AAO using available clinical and familial data on the DIAN cohort. Our assessment focused on large families that include both ADAD mutation carriers and several individuals carrying genome-wide significant risk loci identified in the ADAD GWAS. Only one family (with the ADAD mutation) was informative. We evaluated the impact of the rs537168961 (*CCNG1* locus) risk allele on AAO by comparing the reported AAO of individuals who are both ADAD mutation carriers and rs537168961 (*CCNG1* locus*)* risk allele carriers to the reported AAO of those who carry only the ADAD mutation.

Cerebrospinal fluid biomarker analysis

We utilized the log10-transformed Aβ42/40 ratio in our linear regression analysis.

Neuroimaging analyses

Structural magnetic resonance imaging (MRI) acquisitions were performed on DIAN participants (n=64) in accordance with the Alzheimer's Disease Neuroimaging Initiative (ADNI) protocol.[21,22] These acquisitions included magnetization-prepared rapid gradient echo (MPRAGE) sequences that were subsequently processed with FreeSurfer software (http://surfer.nmr.mgh.harvard.edu/). FreeSurfer analysis involves comprehensive cortical reconstruction and volumetric segmentation of T1-weighted MRI images. This methodology allows for precise delineation of gray-white matter boundaries and pial surfaces, thereby enabling the identification of specific regions of interest (ROIs), including the hippocampus and posterior cingulate cortices. We assessed hippocampal volume (n=63) and cortical thickness in AD signature regions[23] (n=63) using the MRI data. A linear regression adjusted for covariates such as sex and age at the time of the visit was used.

To estimate brain-predicted biological age, whole brain T1 MRIs from DIAN were pre-processed with brain extraction and linear registration, then used as inputs to predict age using the DeepBrainNet (DBN) model.[24] Scans that did not pass QC were excluded. Site/scanner differences in DBN age prediction values were harmonized using ComBat.[25,26] To correct for age-dependent regression-to-the-mean bias, which is common in brain age models (Le et al., 2018), we applied a linear transformation to the harmonized DBN-predicted age values, adjusting for the slope and intercept from a regression model of predicted age as a function of chronological age in healthy control participants.[27,28] The brain age gap was calculated as the difference between harmonized, linear-transformed age prediction from DBN and true chronological age.[29,30] We performed a linear regression analysis to examine the effect of sentinel variants on brain age gap, adjusting for sex as a covariate and corrected with family.

Furthermore, FDG PET was used to assess the whole-brain cerebral metabolic rate for glucose, and Pittsburgh Compound B (PiB) PET was used to obtain measurements of amyloid levels. PiB PET results were quantified using partial volume-corrected standardized uptake value ratios (SUVR) in the gray matter of both the right and left precuneus as defined by FreeSurfer. Additionally, the 18F-FDG PET data were analyzed to determine the average binding potential (BP) across four cortical regions defined by FreeSurfer: the prefrontal cortex, the temporal lobe, the

cingulate gyrus, and the precuneus cortex. We analyzed PET data to measure amyloid levels (n=61) and glucose metabolism (n=59) using a similar model as for MRI measurements. We performed a linear regression analysis as mentioned earlier. Overall, to examine the relationships among these measures and our sentinel SNVs, we applied a Bonferroni correction of $0·05/3=1·7\times10^{-2}$.

2. SUPPLEMENTARY RESULTS

Genome-wide association analysis

To determine if there were any additional underlying signals at each locus, we performed a stepwise conditional analysis on the four identified genome-wide significant loci. Step-wise conditional analysis showed that we did not detect any additional signals within the same locus (figure S5 in appendix). We then performed sensitivity analyses assessing the effect of adding related participants to the cohort and taking into consideration the unbalanced case/control design. Considering the analysis that included related samples, the *ALK* locus was the only locus that did not reach significance ($P=1.51\times10^{-6}$). In addition, while correcting for both relatedness and unbalanced cohort, *RHOJ* was genome-wide significant ($P=1·03\times10^{-8}$), *CNIH4* ($P=2·09\times10^{-6}$) and *CCNG1* ($P=7·36\times10^{-7}$) were nominally significant, and the *ALK* ($P=5.95\times10^{-5}$) locus was not significant. Altogether this confirms that these variants are associated with ADAD risk, even correcting for unbalanced design. Because no significant signal was detected on chromosome 2 in these analyses, we did not consider this locus for follow-up analyses. We also assessed the MAF of the rare allele of the sentinel variants per cohort (figure S6 in appendix). MAF appeared to be consistent throughout the cohorts with no signal driven by any specific cohort. To evaluate whether the three genome-wide significant loci showed consistent effects across causal ADAD genes, we performed stratified association analyses comparing *APP*, *PSEN1*, and *PSEN2* mutation carriers separately against controls. For all three loci, the strongest and most statistically significant effects were observed in *PSEN1* carriers (table S6 in appendix). *APP* and *PSEN2* carriers showed effect estimates in the same direction and of comparable magnitude, although confidence intervals were wide and p-values non-significant, consistent with reduced sample size in these subgroups. These stratified analyses indicate that the associations are not attributed to a single gene, and the direction of effect is consistent across genes.

Impact of sentinel variants on plasma protein levels

We assessed the signals located in the associated region (±500 kb region from the lead variant). The lead GWAS variant was also the lead pQTL variant with no other independent signal detected (figure S8 in Appendix).

Cis-regulatory effect of the sentinel variants

The *RHOJ* had the highest level of expression in astrocytes, endothelial cells, and pericytes in multiple single nucleus RNA-seq datasets from human brain tissues including putamen tissues from individuals with Huntington disease (HD)[31] or controls, entorhinal cortex from individuals with AD[32] , or occipital cortex from Nasu-Hakola disease (NHD)[33] or controls (figure S7 in appendix). Interestingly, *RHOJ* expression was downregulated in multiple astrocyte subpopulations across all three neurodegenerative diseases suggesting astrocytic-specific RHOJ function in neurodegeneration. No other SNVs were located in a cis-regulatory region. Additionally, we assessed the signals located in the associated region (±500 kb region from the lead variant). We detected an independent signal in the eQTL data as compared to the GWAS data, led by rs11624240 (the D' = 0.704 and $r^2 = 0.016$; (figure S10 in Appendix). This suggest that at the RNA level, rs141931440 is not the only variant regulating *RHOJ* expression.

3. SUPPLEMENTARY FIGURES

Figure S1: Identical by descent (IBD) analysis of the participants including in the unrelated Non-Hispanic White study.

X-axis represents the Z0 values, and y-axis represent the Z1 values between individual relationships inference. Z0 values represent the probability that two individuals share 0 alleles identical by descent and typically indicates no recent common ancestry. Z1 values represents the probability that two individuals share 1 allele identical by descent. We removed duplicate and closely related individuals.

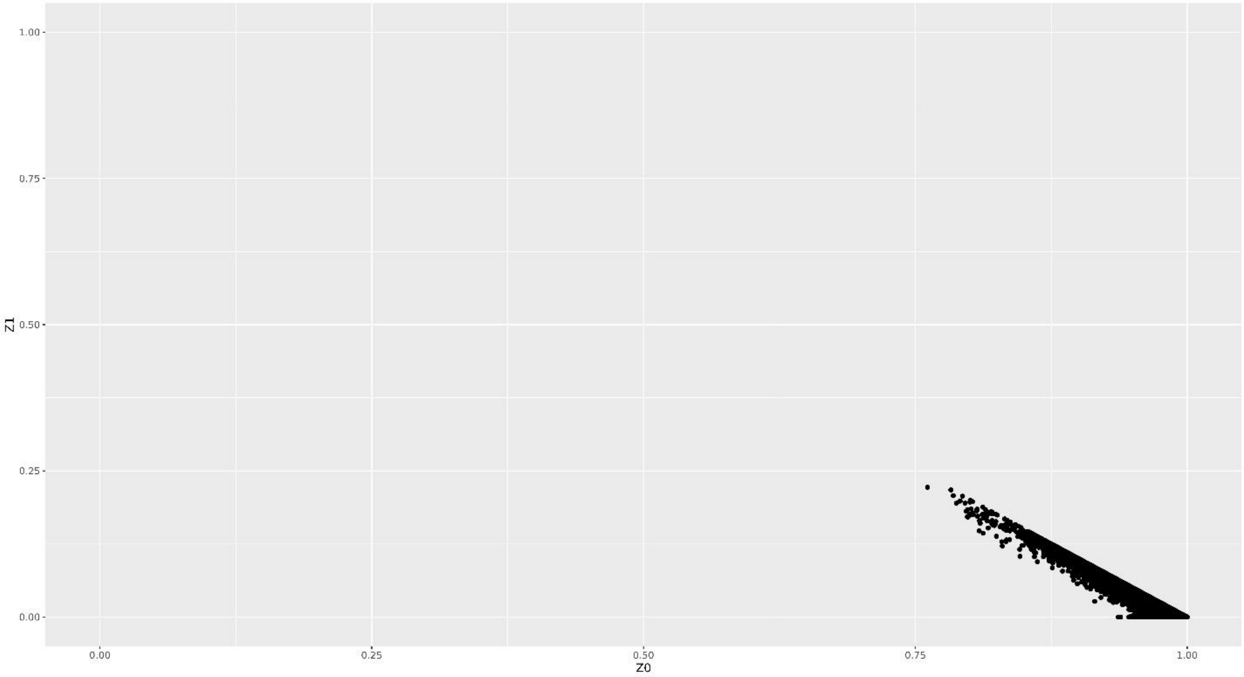

Figure S2: Principal component analysis of case-control participants with 1000 genome as a reference population.

First two principal components (PC1 & PC2) with 1000 genome project as a reference population. AFR: Africans; AMR: Admixed Americans; ASN: Asians; EUR: Europeans; SAN: South Asians. The square block represents the European ancestry included in the study. Participants were selected based on a criterion of being within three standard deviations for the EUR population.

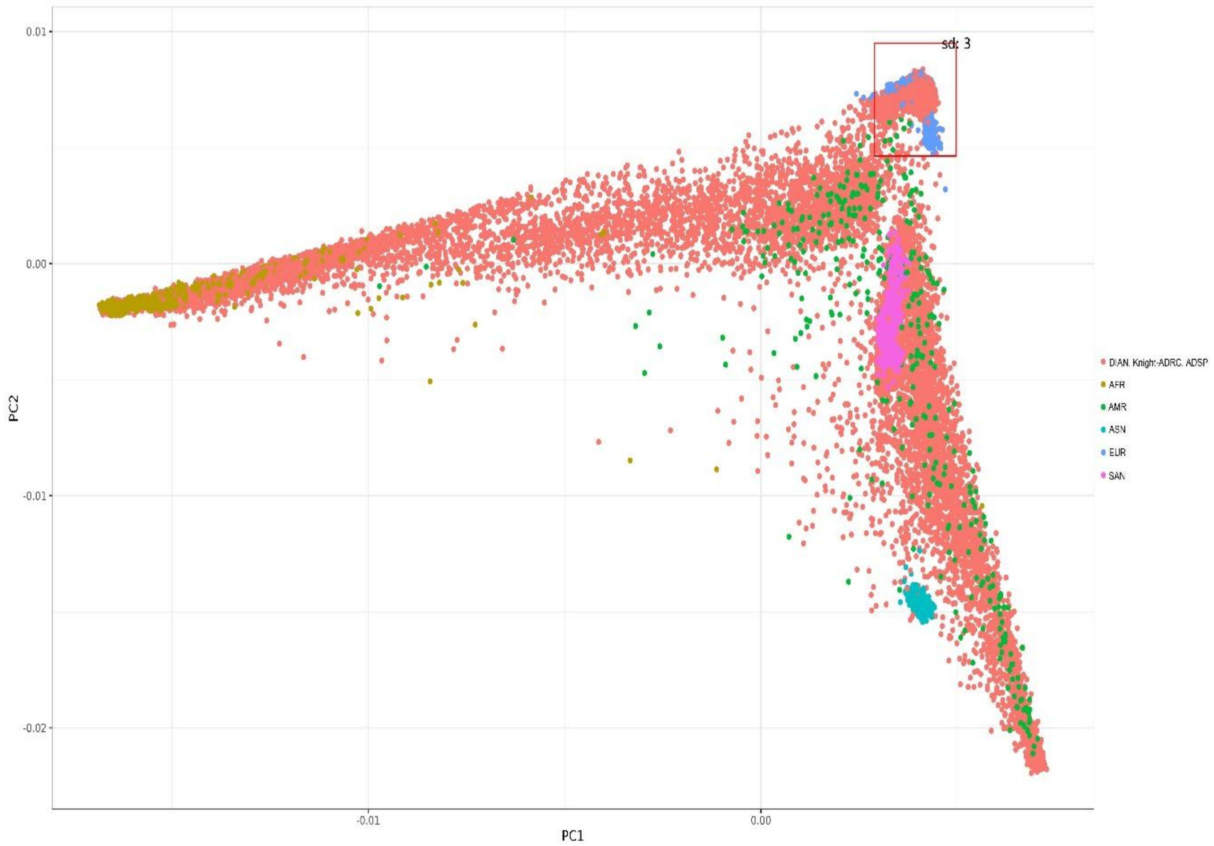

Figure S3: Schematic representation of the sample selection for the ADAD analysis

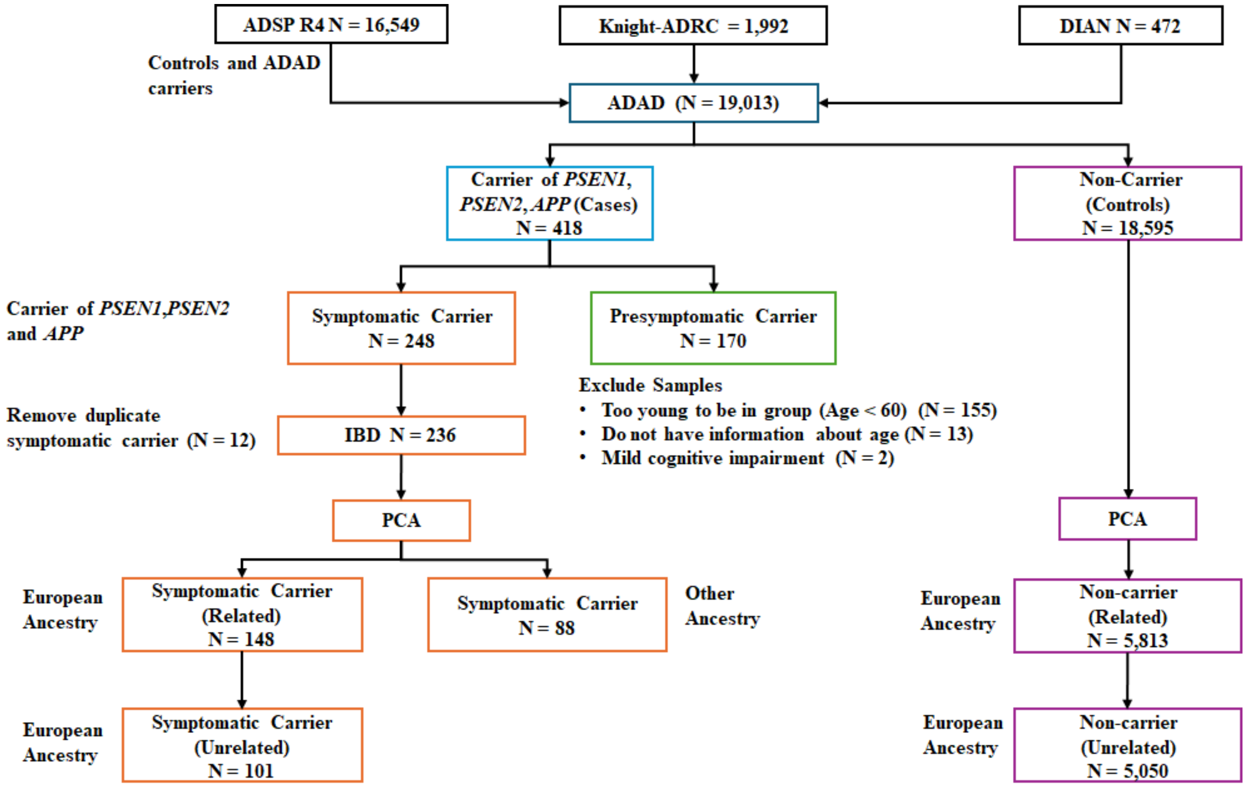

Figure S4: QQ-Plot of genome-wide significant analysis.

The blue points represent observed significance, while the red line denotes the genome-wide significance threshold at a p-value of 5×10^{-8}. A lambda value in a Q-Q plot represents the genomic inflation factor; a value close to 1 indicates no inflation in the test statistics.

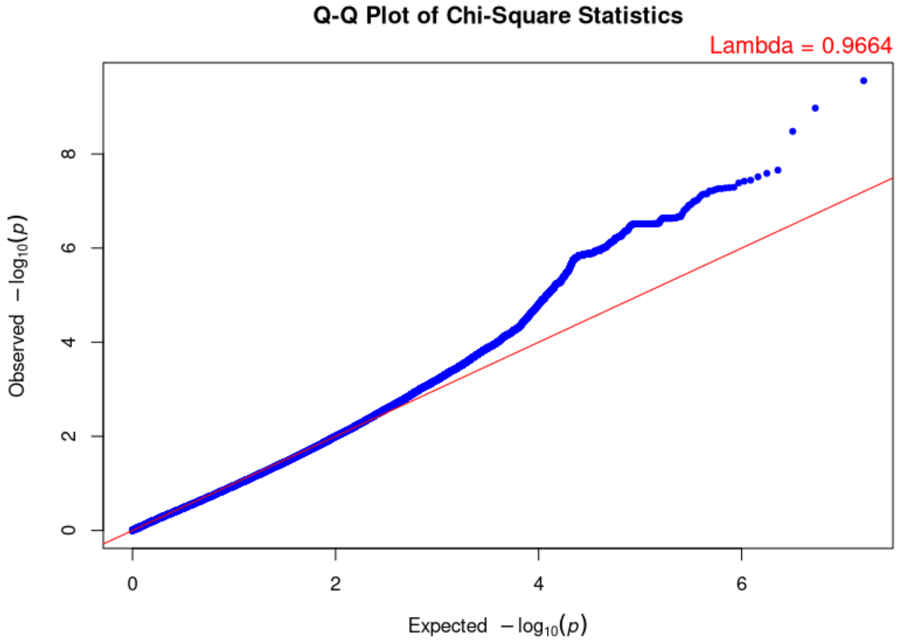

Figure S5: Locuszoom plot of step-wise conditional analysis of the identified sentinel variant of each locus.

A) Conditional analysis at the *CNIH4* locus, conditioning on rs74546314 B) Conditional analysis at the *ALK* locus conditioning on rs78120946 C) Conditional analysis at the *CCNG1* locus conditioning on rs537168961 and, D) Conditional analysis at the *RHOJ* locus conditioning on rs141931440.

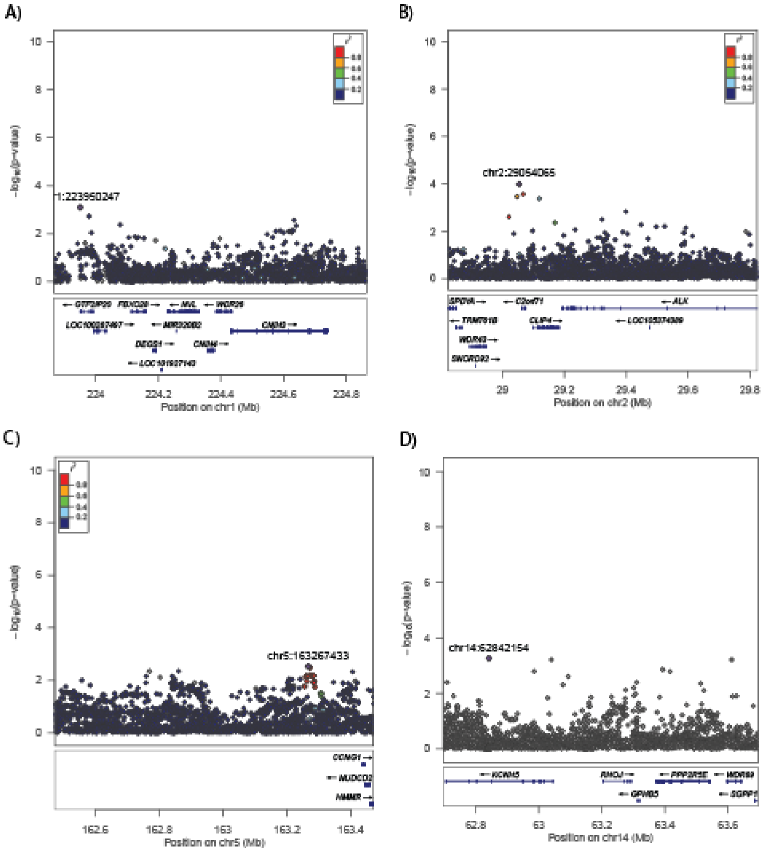

Figure S6: Minor allele frequency of sentinel variants in cases and controls.

A circle (orange color) represents the minor allele frequency (MAF) in cases, while a triangle (blue color) represents the MAF in controls for different cohorts per locus in the study.

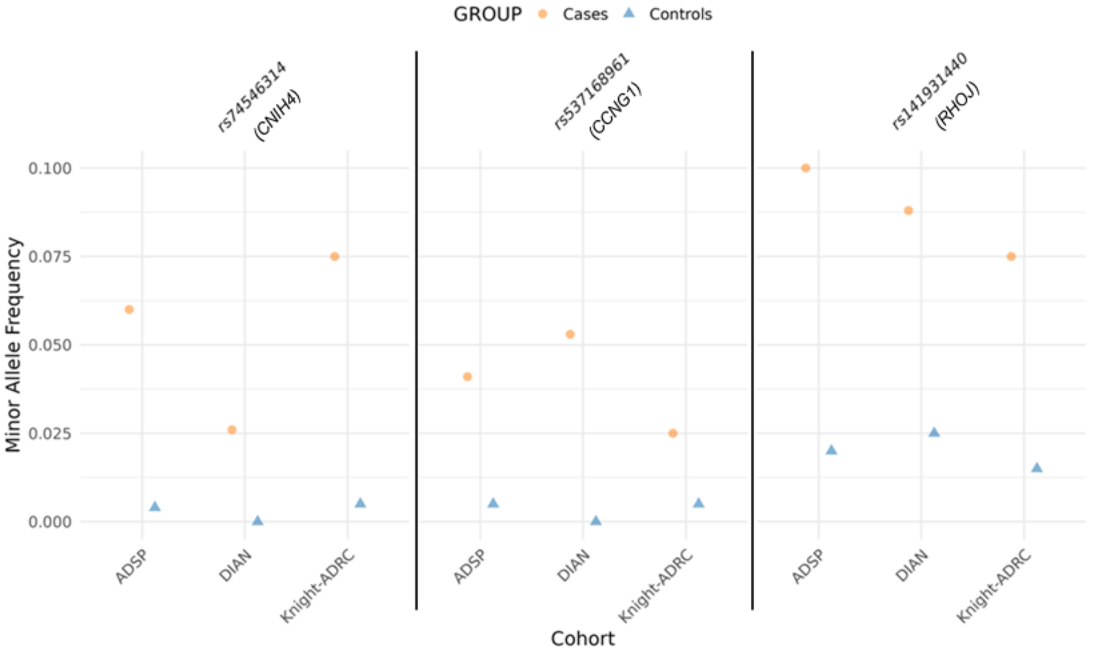

Figure S7: CD33 protein level in plasma among knight-ADRC participants.

CD33 is a pQTL for the RHOJ locus and is negatively correlated with RHOJ. CD33 protein level for the rs141931440 (RHOJ) risk allele carriers and non-carriers among knight-ADRC participants. Dots represent the participants, with orange color for those carrying the rs141931440 risk allele (T) and blue color for those who are homozygous for the rs141931440 reference allele (C). The (TT) represents the homozygous for the (T) allele.

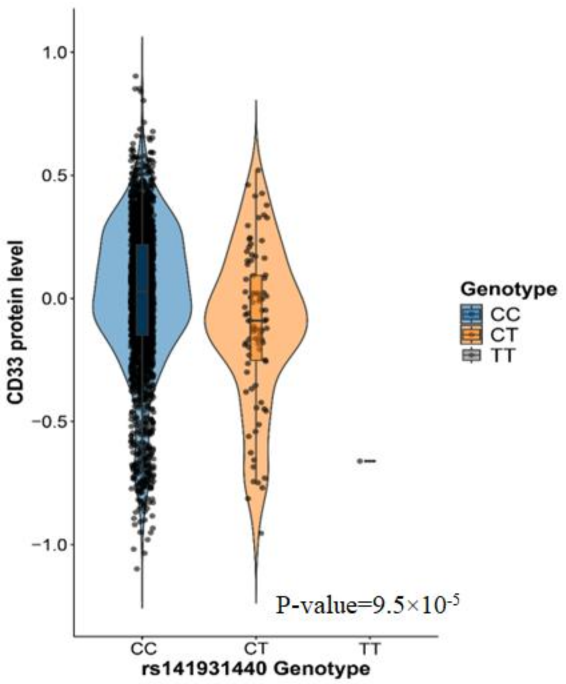

Figure S8: Correlation plot of ADAD GWAS and pQTL datasets

A) Correlation plot between ADAD GWAS (*CCNG1* locus) and pQTL TDP-43. The x-axis represents the -log10 p-values from ADAD GWAS (*CCNG1* locus) and the y-axis represents the -log10 p-values from pQTL TDP-43. Each point in the plot represents a single SNP, colored by the (r^2) value. B) Correlation plot between ADAD GWAS (*RHOJ* locus) and pQTL CD33. The x-axis represents the -log10 p-values from ADAD GWAS (*RHOJ* locus) , and the y-axis represents the -log10 p-values from pQTL CD33.

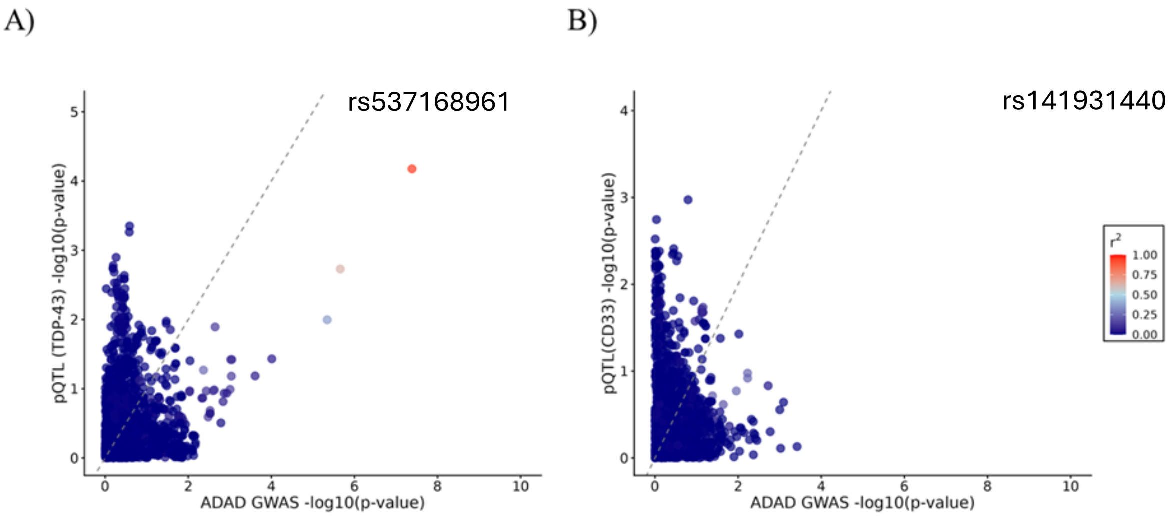

Figure S9: rs141931440 (*RHOJ* locus) is located within a predicted cis-regulatory module.

A) There are two lines of genomic/epigenomic evidence support the functionality of the predicted CRM: a DNAse hypersensitive peak and a H3K27ac peak. B) The results indicate that the carriers of the risk allele at *RHOJ* locus showed downregulated expression in the brain frontal cortex, with a $P=7{\cdot}29\times10^{-3}$ (GTEx database).

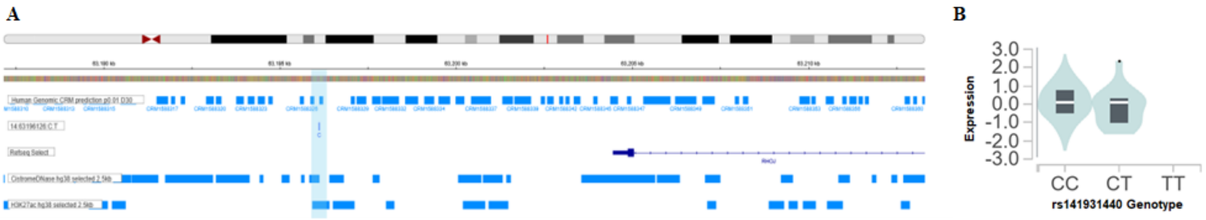

Figure S10: Correlation Plot between ADAD GWAS (rs141931440) and eQTL GTEx brain frontal cortex (BA9) dataset.

The x-axis represents the -log10 p-values from ADAD GWAS, and the y-axis represents the -log10 p-values from GTEx eQTL datasets. Each point in the plot represents a single SNP, colored by the (r^2) value.

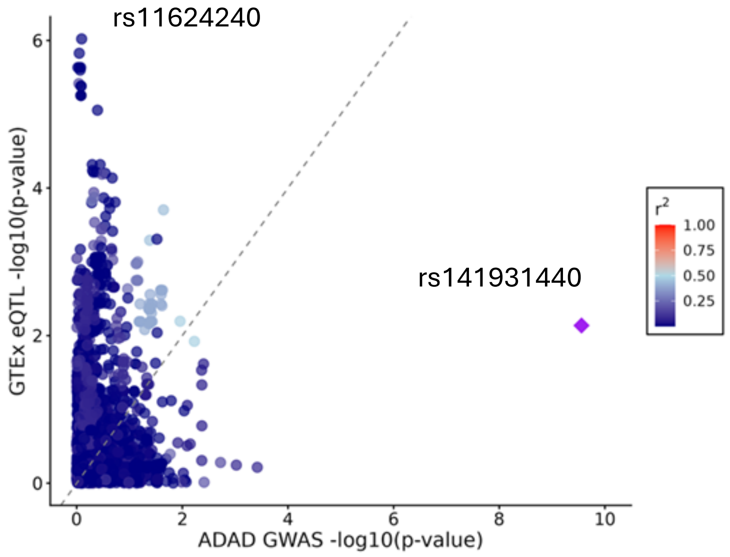

Figure S11: Differential expression of the *RHOJ* gene in astrocytes under various disease conditions.

In this figure, various cell types are analyzed across different datasets, including Astrocytes (Ast), Endothelial Cells (ENDO), Endothelial Cells and Pericytes (EP), Microglial immune cells (Microglia), Neuronal Cell Types (Neuron), Oligodendrocytes (OLIGO), Oligodendrocyte Precursor Cells (OPC), Pericytes, and T Cells. *RHOJ* expression levels in various cell types in **A)** Huntington's disease (HD), **B)** Alzheimer's disease (AD), and **C)** Nasu-Hakola disease (NHD). Comparison of *RHOJ* expression levels in control vs. HD, AD, and NHD, illustrating that *RHOJ* expression is downregulated in astrocyte cell types in AD.

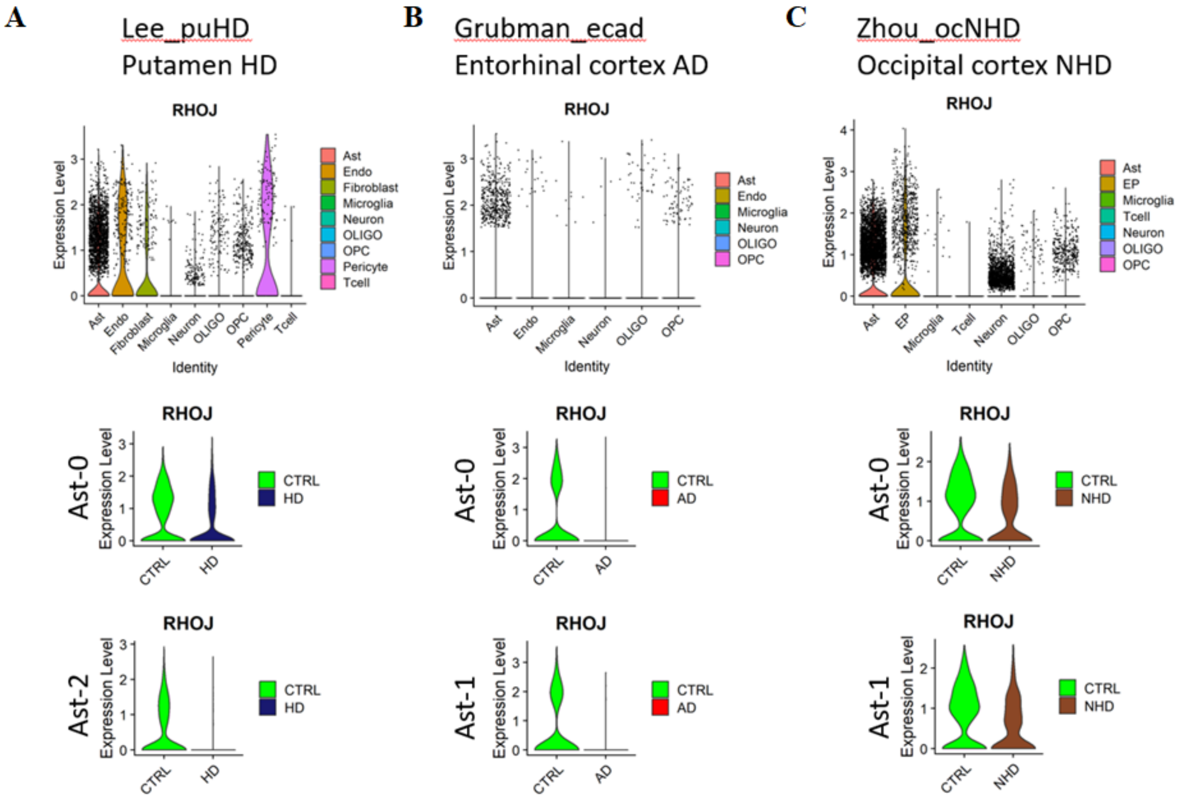

Figure S12: Age at onset distribution of carriers and non-carriers of the *CCNG1* sentinel (rs537168961) risk allele.

Light orange color represents the carrier of the rs537168961 risk allele and light blue color represents the non-carrier of rs537168961 risk allele.

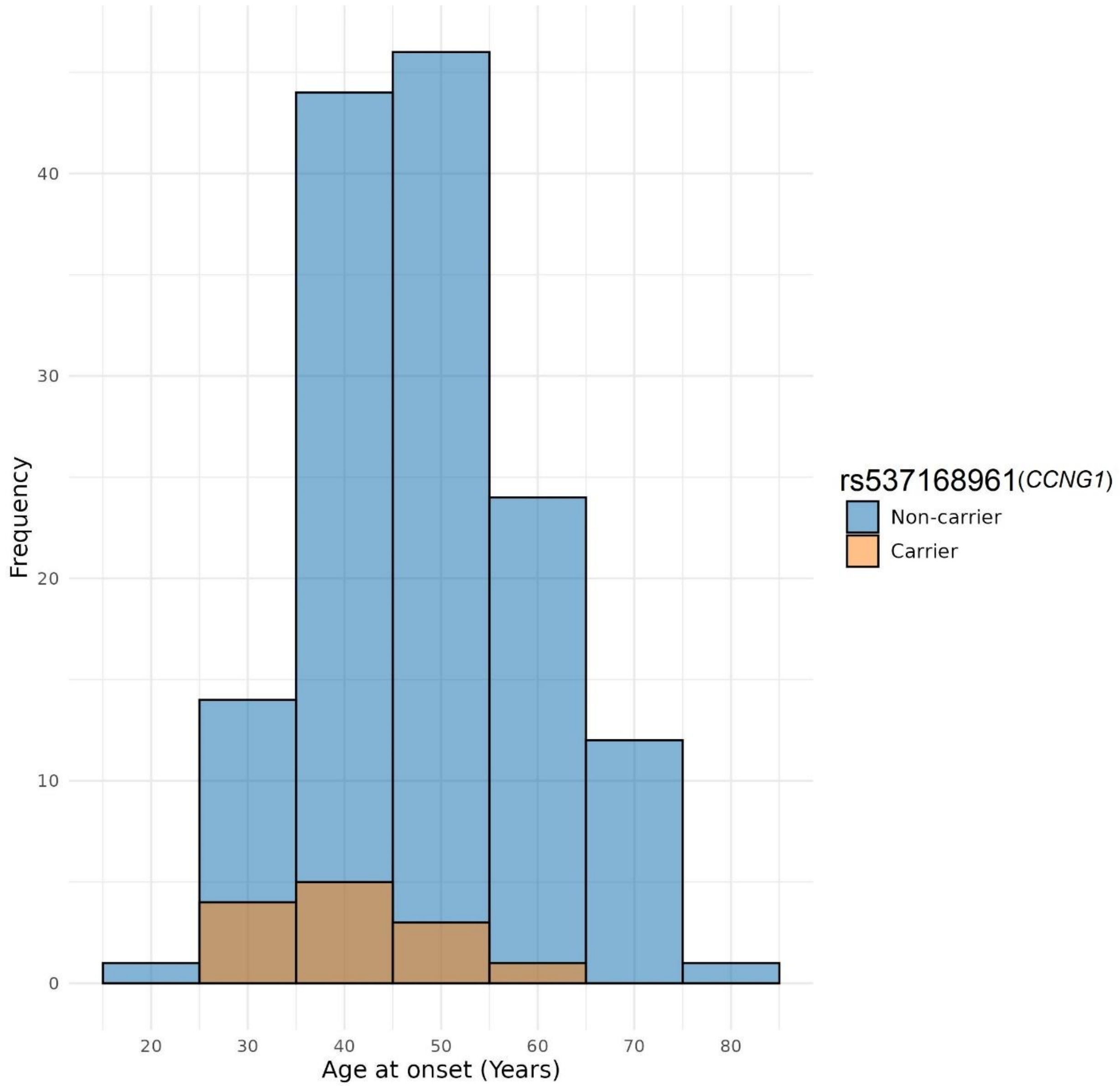

4. SUPPLEMENTARY TABLES

Table S1: List of mutations in *APP*, *PSEN2* and *PSEN1* genes.

Protein Change= Variant nomenclature based on protein Human Genome Variation Society; Transcript= Ensemble transcript ID; Chromosome:Position: Chromosome and position of the variant; rsIDs= variant ID from dbSNP; N total participant= Number of participants; N participant (NHW) = Number of NHW participants included in the unrelated GWAS analysis; CADD= Predictive pathogenic score of the variant; Clinical Significance= variant is pathogenic or likely pathogenic based on ALZFORUM, DIAN Mutation database and Clinvar clinical significance.

Gene Name	Protein change	Transcript	Chromosome:Position	rsIDs	N Total Participant	N NHW Participant	CADD	Clinical Significance
APP	p.Asn660Tyr	ENST00000346798.8	chr21:25897659:T:A	–	< 3	< 3	25·2	Pathogenic
	p.Met671Leu	ENST00000346798.8	chr21:25897626:T:G	rs572842823	4	< 3	22·6	Pathogenic
	p.Glu693Gln	ENST00000346798.8	chr21:25891856:C:G	rs63750579	10	< 3	27·4	Pathogenic
	p.Asp694Asn	ENST00000346798.8	chr21:25891853:G:A	rs63749810	< 3	< 3	27·4	Pathogenic
	p.Ile716Val	ENST00000346798.8	chr21:25891787:T:C	rs63750399	4	< 3	26·0	Pathogenic
	p.Val715Ala	ENST00000346798.8	chr21:25891789:A:G	rs63750868	< 3	NA	28·1	Pathogenic
	p.Val717Gly	ENST00000346798.8	chr21:25891783:T:G	rs63749964	< 3	< 3	25·8	Pathogenic
	p.Val717Ile	ENST00000346798.8	chr21:25891784:C:T	rs63750264	29	9	24·2	Pathogenic
	p.Val717Leu	ENST00000346798.8	chr21:25891784:C:G	rs63750264	3	NA	26·4	Pathogenic
PSEN2	p.Ser175Cys	ENST00000366782.6	chr1:226888116:A:C	rs775145486	< 3	< 3	29·5	Likely Pathogenic
	p.Asn141Ile	ENST00000366782.6	chr1:226885603:A:T	rs63750215	26	5	26·9	Pathogenic
PSEN1	p.Ala79Val	ENST00000324501.10	chr14:73170945:C:T	rs63749824	22	10	26·3	Pathogenic
	p.Met84Val	ENST00000324501.10	chr14:73170959:A:G	–	< 3	NA	23·9	Pathogenic
	p.Phe105Leu	ENST00000324501.10	chr14:73171024:T:G	rs63750321	< 3	< 3	23·6	Pathogenic
	p.Phe105Ser	ENST00000324501.10	chr14:73171023:T:C	–.	3	< 3	29·0	Pathogenic
	c.338+7A>G	ENST00000324501.10	chr14:73171053:A:G	–	4	NA	·	Likely Pathogenic
	p.Tyr115Cys	ENST00000324501.10	chr14:73173571:A:G	rs63750450	< 3	< 3	29·9	Pathogenic
	p.Tyr115His	ENST00000324501.10	chr14:73173570:T:C	–	3	< 3	29·0	Pathogenic
	p.Thr119Ile	ENST00000324501.10	chr14:73173583:C:T	rs1566630791	< 3	< 3	24·1	Pathogenic

p.Asn135Tyr	ENST00000324501.10	chr14:73173630:A:T	rs63750353	< 3	< 3	29·8	Pathogenic
p.Asn135Ser	ENST00000324501.10	chr14:73173631:A:G	rs63751278	3	NA	26·9	Pathogenic
p.Met139Ile	ENST00000324501.10	chr14:73173644:G:A	rs63750522	< 3	NA	25·0	Pathogenic
p.Met139Val	ENST00000324501.10	chr14:73173642:A:G	rs63751037	4	4	23·9	Pathogenic
p.Ile143Thr	ENST00000324501.10	chr14:73173655:T:C	rs63750004	< 3	< 3	27·5	Pathogenic
p.Met146Ile	ENST00000324501.10	chr14:73173665:G:A	rs63750391	3	NA	28·7	Pathogenic
p.Met146Leu	ENST00000324501.10	chr14:73173663:A:T	rs63750306	4	< 3	26·1	Pathogenic
p.Met146Val	ENST00000324501.10	chr14:73173663:A:G	rs63750306	< 3	NA	25·2	Pathogenic
p.His163Arg	ENST00000324501.10	chr14:73186860:A:G	rs63750590	27	8	25·2	Pathogenic
p.Ser169Leu	ENST00000324501.10	chr14:73186878:C:T	rs63751210	3	< 3	29·1	Pathogenic
p.Ser170Phe	ENST00000324501.10	chr14:73186881:C:T	rs63750577	< 3	< 3	28·2	Pathogenic
p.Phe175Leu	ENST00000324501.10	chr14:73186897:C:A	–	3	< 3	24·6	Pathogenic
p.Ser178Pro	ENST00000324501.10	chr14:73186904:T:C	rs63750155	4	< 3	26·5	Pathogenic
p.Glu184Asp	ENST00000324501.10	chr14:73192647:A:C	rs63750311	6	< 3	24·2	Pathogenic
p.Gly206Ala	ENST00000324501.10	chr14:73192712:G:C	rs63750082	75	NA	26·6	Pathogenic
p.Gly209Glu	ENST00000324501.10	chr14:73192721:G:A	–	3	< 3	27·6	Pathogenic
p.Ser212Tyr	ENST00000324501.10	chr14:73192730:C:A	rs1555355250	5	< 3	23·7	Pathogenic
p.Gly217Arg	ENST00000324501.10	chr14:73192744:G:C	rs267606983	5	< 3	28·5	Pathogenic
p.Leu226Arg	ENST00000324501.10	chr14:73192772:T:G	rs63749961	< 3	NA	28·9	Pathogenic
p.Ile229Phe	ENST00000324501.10	chr14:73192780:A:T	rs63749970	< 3	< 3	27·6	Pathogenic
p.Ser230Asn	ENST00000324501.10	chr14:73192784:G:A	rs1555355284	< 3	< 3	26·8	Pathogenic
p.Ala231Thr	ENST00000324501.10	chr14:73192786:G:A	rs63749836	< 3	< 3	26·5	Likely Pathogenic
p.Met233Leu	ENST00000324501.10	chr14:73192792:A:C	–	< 3	< 3	26·1	Pathogenic
p.Leu235Val	ENST00000324501.10	chr14:73192798:C:G	rs63751130	4	< 3	23·3	Likely Pathogenic
p.Ile238Met	ENST00000324501.10	chr14:73192809:C:G	rs1555355289	3	< 3	24·4	Likely Pathogenic
p.Thr245Pro	ENST00000324501.10	chr14:73192828:A:C	rs63750888	< 3	NA	25·1	Likely Pathogenic
p.Ile249Leu	ENST00000324501.10	chr14:73192840:A:C	rs1362575880	3	NA	24·5	Pathogenic
p.Ala260Gly	ENST00000324501.10	chr14:73198040:C:G	–	10	< 3	28·6	Likely Pathogenic
p.Val261Phe	ENST00000324501.10	chr14:73198042:G:T	rs63750964	< 3	< 3	31·0	Likely Pathogenic
p.Pro264Leu	ENST00000324501.10	chr14:73198052:C:T	rs63750301	10	6	26·8	Pathogenic

p.Gly266Ser	ENST00000324501.10	chr14:73198057:G:A	rs121917807	< 3	< 3	25·9	Pathogenic
p.Arg269His	ENST00000324501.10	chr14:73198067:G:A	rs63750900	14	4	32·0	Likely Pathogenic
p.Leu271Val	ENST00000324501.10	chr14:73198072:C:G	rs63750886	6	< 3	23·9	Pathogenic
p.Glu273Lys	ENST00000324501.10	chr14:73198078:G:A	–	5	< 3	32·0	Pathogenic
p.Ala275Val	ENST00000324501.10	chr14:73198085:C:T	rs1555355869	< 3	< 3	27·6	Pathogenic
p.Glu280Gly	ENST00000324501.10	chr14:73198100:A:G	rs63750231	4	< 3	29·0	Pathogenic
p.Phe283Leu	ENST00000324501.10	chr14:73198108:T:C	–	< 3	NA	28·8	Pathogenic
p.Tyr288His	ENST00000324501.10	chr14:73198123:T:C	–	4	< 3	29·2	Pathogenic
p.Phe386Ile	ENST00000324501.10	chr14:73217152:T:A	–	< 3	NA	26·4	Pathogenic
p.Gly378Val	ENST00000324501.10	chr14:73217129:G:T	rs63750323	< 3	< 3	33·0	Pathogenic
p.Ala396Thr	ENST00000324501.10	chr14:73217182:G:A	–	< 3	< 3	27·0	Likely Pathogenic
p.Cys410Tyr	ENST00000324501.10	chr14:73217225:G:A	rs661	7	3	29·5	Pathogenic
p.Ala426Pro	ENST00000324501.10	chr14:73219161:G:C	rs63751223	21	3	31·0	Pathogenic
p.Ala431Glu	ENST00000324501.10	chr14:73219177:C:A	rs63750083	20	NA	26·3	Pathogenic
c.869-1G>T	ENST00000324501.10	chr14:73673093:G:T	–	5	3	–	Pathogenic
c.869-1G>A	ENST00000324501.10	chr14:73673093:G:A	–	5	< 3	–	Pathogenic

Table S2: Demographic information

This table summarizes the information of participants included in case-control GWAS analysis, including related participants. Participants (Female %)= Number of participants and % of females; median age (IQR)= median age at last healthy visit for controls and median age at onset for cases participants. IQR= Interquartile range. *APOE ε4(%):* participants has one or two copies of the *APOE ε4* and their percentage

		**Participants (Female %)**	**Median age (IQR)**	***APOE ε4 (%)***
**Control**		5,813 (60·19)	79·00 (73·00 – 85·00)	1633 (28·09)
**Cases**		148 (46·62)	47·00 (40·00 – 54·00)	51 (34·45)
	**ADAD genes**			
	APP	21 (66·66)	46·00 (40·00 – 53·00)	12 (57·14)
	PSEN1	118 (42·37)	46·00 (39·00 – 53·50)	32 (27·11)
	PSEN2	9 (55·56)	46·00 (40·00 – 53·00)	7 (77·77)

Table S3: Genome-wide significant loci with and without adjusting for *APOE ε4*

This table summarizes the information of genome-wide significant loci ($P<5{\cdot}0\times10^{-8}$) identified in case-control GWAS analysis. Locus: Nearest locus gene name; rsID: dbSNP reported variant ID; SNV: chromosome (GRCh38), base-pair position, Reference allele, Alternative allele; P without *APOE ε4*: P-value without adjusting *APOE ε4*; OR: Odds ratio; CI: Confidence Interval; P with *APOE ε4*: P-value with adjusting *APOE ε4*

**Locus**	**rsID**	**SNV**	**P without *APOE ε4***	**OR (CI)**	**P with *APOE ε4***	**OR (CI)**
CNIH4	rs74546314	1:224365900:G:A	$1{\cdot}05\times10^{-9}$	11·99 (5·39 – 26·64)	$7{\cdot}42\times10^{-10}$	12·61 (5·62 – 28·26)
CCNG1	rs537168961	5:162973378:C:T	$3{\cdot}05\times10^{-8}$	9·56 (4·29 – 21·24)	$1{\cdot}63\times10^{-8}$	10·05 (4·51 – 22·41)
RHOJ	rs141931440	14:63196126:C:T	$2{\cdot}77\times10^{-10}$	5·96 (3·42 – 10·36)	$9{\cdot}29\times10^{-11}$	6·32 (3·61 – 11·04)

Table S4: Sex stratified analysis (case/control association study) for the genome-wide significant sentinel variants.

Locus: Nearest locus gene name; rsID: dbSNP reported variant ID; SNV: chromosome(GRCh38), base-pair position, Reference allele; Alternative allele; OR: Odds Ratio; CI: Confidence Interval; P: P-value

Locus	rsID	SNV	Male		Female	
			OR (CI)	P	OR (CI)	P
CNIH4	rs74546314	1:224365900:G:A	11·01 (3·39 – 35·73)	$6{\cdot}51\times10^{-5}$	15·00 (4·83 – 46·60)	$2{\cdot}80\times10^{-6}$
ALK	rs78120946	2:29324788:T:A	7·87 (3·19 – 19·38)	$7{\cdot}26\times10^{-6}$	6·95 (2·19 – 22·03)	$9{\cdot}85\times10^{-4}$
CCNG1	rs537168961	5:162973378:C:T	14·10 (4·47 – 44·48)	$6{\cdot}33\times10^{-6}$	6·08 (1·88 – 19·59)	$2{\cdot}49\times10^{-3}$
RHOJ	rs141931440	14:63196126:C:T	5·11 (2·37 – 11·01)	$3{\cdot}01\times10^{-5}$	6·72 (2·97 – 15·23)	$4{\cdot}84\times10^{-6}$

Table S5: Carriers of sentinel variants and their ADAD mutations.

This table summarizes the information on carriers of genome-wide significant loci, including details for both cases and controls. Additionally, it provides per-gene information for case carriers, including the percentage of females and unique mutations for each chromosome locus. Carrier N= Number of carrier per loci with percentage of female, Unique Mutation= No of unique mutations.

	rs74546314		rs537168961		rs141931440	
	**Carriers N (%female)**	**Unique mutations**	**Carriers N (%female)**	**Unique mutations**	**Carriers N (%female)**	**Unique mutations**
Control	45 (62·22)	–	53 (71·70)	–	212 (57·08)	–
Cases	9 (55·56)	4	9 (44·44)	8	18 (50·00)	11
PSEN1	2 (0·00)	2	8 (50·00)	7	15 (46·67)	9
PSEN2	7 (71·43)	2	–	–	1 (0·00)	1
APP	–	–	1	1	2 (100)	1

Table S6: Association study stratified by ADAD genes for sentinel variants.

This table summarizes the information of stratified analysis by ADAD genes for sentinel variants. Locus: Nearest locus gene name; rsID: dbSNP reported variant ID; OR: Odds Ratio; CI: Confidence Interval; P: P-value.

Locus	rsID	*APP* Cases/Controls		*PSEN1* Cases/Controls		*PSEN2* Cases/Controls	
		P	OR (CI)	P	OR (CI)	P	OR (CI)
CNIH4	rs74546314	$4{\cdot}61\times10^{-1}$	2·82 (0·17 – 44·74)	$1{\cdot}23\times10^{-1}$	3·16 (0·72 – 13·70)	NA	NA
CCNG1	rs537168961	$7{\cdot}01\times10^{-2}$	7·01 (0·80 – 61·19)	$1{\cdot}51\times10^{-8}$	11·40 (4·90 – 26·48)	$1{\cdot}95\times10^{-1}$	5·94 (0·39 – 88·60)
RHOJ	rs141931440	$5{\cdot}41\times10^{-2}$	4·63 (0·97 – 22·07)	$1{\cdot}09\times10^{-9}$	6·53 (3·57 – 11·94)	$1{\cdot}53\times10^{-1}$	4·86 (0·55 – 42·75)

Table S7: Association analysis stratified by ADAD gene with *APOE ε4* interaction term.

Locus: Nearest locus gene name; rsID: dbSNP reported variant ID; Beta (CI): Effect size (Confidence Interval); P: P-value

**Locus**	**rsID**	***APP* Cases/Controls**		***PSEN1* Cases/Controls**		***PSEN2* Cases/Controls**	
		**P**	**OR (CI)**	**P**	**OR (CI)**	**P**	**OR (CI)**
CNIH4	rs74546314	$9{\cdot}93\times10^{-1}$	NA	$5{\cdot}89\times10^{-2}$	4·18 (0·96 – 18·18)	$9{\cdot}96\times10^{-1}$	NA
CCNG1	rs537168961	$3{\cdot}87\times10^{-2}$	10·38 (1·13 – 95·43)	$9{\cdot}12\times10^{-9}$	14·46 (5·86 – 35·56)	$9{\cdot}93\times10^{-1}$	NA
RHOJ	rs141931440	$2{\cdot}91\times10^{-2}$	5·70 (1·19 – 27·36)	$1{\cdot}72\times10^{-7}$	6·17 (3·11 – 12·27)	$9{\cdot}94\times10^{-1}$	NA

Table S8: Replication and meta-analysis using ADSP R5 datasets.

This table summarizes the information of replication analysis using ADSP R5 NHW case-control GWAS analysis. Locus: Nearest locus gene name; rsID: Single nucleotide reported variant ID; SNV: chromosome: GRCh38 base-pair position: Reference Allele; Alternate Allele; P: P-value; OR: Odds ratio; CI: Confidence Interval; Discovery MAF: Minor allele frequency of discovery study; Replication MAF: Minor allele frequency of replication study; gnomAD MAF: Minor allele frequency in gnomAD non-finish European

Locus	rsID	SNV	Replication		Meta-analysis		Discovery MAF	Replication MAF	gnomAD MAF
			P	OR (CI)	P	OR (CI)			
CNIH4	rs74546314	1:224365900:G:A	$7{\cdot}30\times10^{-1}$	1·60 (0·10 – 23·38)	$2{\cdot}77\times10^{-9}$	10·16 (4·73 – 21·84)	0·005	0·002	0·003
CCNG1	rs537168961	5:162973378:C:T	$5{\cdot}76\times10^{-1}$	1·75 (0·24 – 12·53)	$9{\cdot}22\times10^{-8}$	7·47 (3·61 – 15·41)	0·006	0·004	0·008
RHOJ	rs141931440	14:63196126:C:T	$3{\cdot}64\times10^{-2}$	3·01 (1·07 – 8·46)	$5{\cdot}74\times10^{-11}$	5·11 (3·13 – 8·34)	0·022	0·019	0·018

Table S9: Case/Control GWAS analysis of sporadic AD using ADSP datasets.

This table summarizes the information of our identified ADAD GWAS significant loci in ADSP case-control sporadic AD GWAS analysis. Locus: Nearest locus gene name; rsID: Single nucleotide reported variant ID; SNV: chromosome: GRCh38 base-pair position: Reference Allele; Alternate Allele; P: P-value; OR: Odds ratio; CI: Confidence Interval.

Locus	rsID	SNV	P	OR (CI)
CNIH4	rs74546314	1:224365900:G:A	$4{\cdot}47\times10^{-1}$	0·85 (0·55 – 1·29)
CCNG1	rs537168961	5:162973378:C:T	$6{\cdot}53\times10^{-2}$	1·39 (0·97 – 1·98)
RHOJ	rs141931440	14:63196126:C:T	$2{\cdot}26\times10^{-1}$	0·89 (0·73 – 1·07)

Table S10: Plasma pQTL analysis of risk loci

pQTL (Plasma)= Protein target name for that locus in plasma; rsID= Single nucleotide reported variant ID; P= P-value; Beta= effect size for that Locus; CI= confidence Interval. These data include Knight-ADRC participants, which include AD patients, ADAD, healthy controls, frontotemporal dementia patients, and individuals with an unclassified neurodegenerative disease.

SNV	rsID	Locus	pQTL (plasma)	P	Beta (CI)
5:162973378:C:T	rs537168961	*CCNG1*	TDP-43	$9{\cdot}00\times10^{-6}$	0·09 (0·04 – 0·12)
14:63196126:C:T	rs141931440	*RHOJ*	CD33	$9{\cdot}50\times10^{-5}$	-0·13 (-0·19 – -0·06)

Table S11: Sex stratified analysis on age at onset (AAO) of genome-wide significant loci.

Locus: Nearest locus gene name; rsID: dbSNP reported variant ID; SNV: chromosome(GRCh38), base-pair position, Reference allele; Alternative allele; Beta (CI): Effect size (Confidence Interval); P: P-value

Locus	rsID	SNV	Male		Female	
			P	Beta (CI)	P	Beta (CI)
CNIH4	rs74546314	1:224365900:G:A	5·09×10^{-1}	4·00 (-7·74 – 15·74)	5·03×10^{-1}	3·97 (-7·54 – 15·50)
CCNG1	rs537168961	5:162973378:C:T	1·20×10^{-1}	-10·43 (6·52 – -23·23)	1·36×10^{-1}	-8·78 (5·74 – -20·05)
RHOJ	rs141931440	14:63196126:C:T	7·19×10^{-1}	-1·81 (-11·61 – 7·99)	6·85×10^{-1}	2·29 (-8·73 – 13·33)

Table S12: Linear regression analysis with Lumipulse CSF AD biomarkers: CSF tTAU, CSF pTau181, and CSF Aβ42/40.

CSF tTAU: CSF total Tau; CSF pTau181: CSF phosphorylated Tau protein; CSF Aβ42/40: Aβ 42 and Aβ 40 ratio rsID:variant ID from dbSNP; P: P-value; Beta: effect size; SE: Standard Error. In this analysis, we included related NHW ADAD cases participants.

SNV	rsID	Locus	CSF tTau		CSF pTau181		CSF Aβ42/40	
			P	Beta (SE)	P	Beta (SE)	P	Beta (SE)
1:224365900:G:A	rs74546314	*CNIH4*	$4·85\times10^{-1}$	-165·92 [236·24]	$2·18\times10^{-1}$	-55·17 [44·32]	$1·67\times10^{-1}$	0·02 [0·01]
5:162973378:C:T	rs537168961	*CCNG1*	$7·15\times10^{-2}$	58·42 [159·33]	$4·48\times10^{-1}$	22·45 [29·36]	$3·86\times10^{-1}$	0·01 [0·01]
14:63196126:C:T	rs141931440	*RHOJ*	$5·55\times10^{-3}$	358·37 [124·21]	$6·46\times10^{-4}$	81·28 [22·56]	$1·57\times10^{-2}$	-0·11 [0·01]

Table S13: Demographics of participants with and without neuroimaging

Demographic Variable	With Imaging (n=64)	Without Imaging (n=84)	Overall Study Population (n=148)
Median age (IQR)	45 (39-50)	49 (42-59)	47 (40-54)
Sex (% female)	56.25%	39.28%	46.62%

5. References:

1. Li H, Durbin R. Fast and accurate short read alignment with Burrows-Wheeler transform. Bioinformatics 2009; 25(14): 1754-60.

2. McKenna A, Hanna M, Banks E, et al. The Genome Analysis Toolkit: a MapReduce framework for analyzing next-generation DNA sequencing data. Genome Res 2010; 20(9): 1297-303.

3. Li H. A statistical framework for SNP calling, mutation discovery, association mapping and population genetical parameter estimation from sequencing data. Bioinformatics 2011; 27(21): 2987-93.

4. Leung YY, Lee WP, Kuzma AB, et al. Alzheimer's Disease Sequencing Project Release 4 Whole Genome Sequencing Dataset. medRxiv 2024.

5. Purcell S, Neale B, Todd-Brown K, et al. PLINK: a tool set for whole-genome association and population-based linkage analyses. Am J Hum Genet 2007; 81(3): 559-75.

6. Manichaikul A, Mychaleckyj JC, Rich SS, Daly K, Sale M, Chen WM. Robust relationship inference in genome-wide association studies. Bioinformatics 2010; 26(22): 2867-73.

7. Cruchaga C, Heo G, Thomas A, et al. Large-scale Plasma Proteomic Profiling Unveils Novel Diagnostic Biomarkers and Pathways for Alzheimer's Disease. Res Sq 2025.

8. Yang C, Farias FHG, Ibanez L, et al. Genomic atlas of the proteome from brain, CSF and plasma prioritizes proteins implicated in neurological disorders. Nat Neurosci 2021; 24(9): 1302-12.

9. Timsina J, Gomez-Fonseca D, Wang L, et al. Comparative Analysis of Alzheimer's Disease Cerebrospinal Fluid Biomarkers Measurement by Multiplex SOMAscan Platform and Immunoassay-Based Approach. J Alzheimers Dis 2022; 89(1): 193-207.

10. Cruchaga C, Western D, Timsina J, et al. Proteogenomic analysis of human cerebrospinal fluid identifies neurologically relevant regulation and informs causal proteins for Alzheimer's disease. Res Sq 2023.

11. Cruchaga C, Yang C, Gorijala P, et al. European and African-specific plasma protein-QTL and metabolite-QTL analyses identify ancestry-specific T2D effector proteins and metabolites. Res Sq 2024.

12. Visel A, Minovitsky S, Dubchak I, Pennacchio LA. VISTA Enhancer Browser--a database of tissue-specific human enhancers. Nucleic Acids Res 2007; 35(Database issue): D88-92.

13. Consortium EP, Moore JE, Purcaro MJ, et al. Expanded encyclopaedias of DNA elements in the human and mouse genomes. Nature 2020; 583(7818): 699-710.

14. Zhang K, Hocker JD, Miller M, et al. A single-cell atlas of chromatin accessibility in the human genome. Cell 2021; 184(24): 5985-6001 e19.

15. Consortium GT. The Genotype-Tissue Expression (GTEx) project. Nat Genet 2013; 45(6): 580-5.

16. Wingo AP, Liu Y, Gerasimov ES, et al. Integrating human brain proteomes with genome-wide association data implicates new proteins in Alzheimer's disease pathogenesis. Nat Genet 2021; 53(2): 143-6.

17. Ng B, White CC, Klein HU, et al. An xQTL map integrates the genetic architecture of the human brain's transcriptome and epigenome. Nat Neurosci 2017; 20(10): 1418-26.

18. Gagliano Taliun SA, VandeHaar P, Boughton AP, et al. Exploring and visualizing large-scale genetic associations by using PheWeb. Nat Genet 2020; 52(6): 550-2.

19. de Klein N, Tsai EA, Vochteloo M, et al. Brain expression quantitative trait locus and network analyses reveal downstream effects and putative drivers for brain-related diseases. Nat Genet 2023; 55(3): 377-88.

20. Dudley WN, Wickham R, Coombs N. An Introduction to Survival Statistics: Kaplan-Meier Analysis. J Adv Pract Oncol 2016; 7(1): 91-100.

21. McKay NS, Gordon BA, Hornbeck RC, et al. Positron emission tomography and magnetic resonance imaging methods and datasets within the Dominantly Inherited Alzheimer Network (DIAN). Nat Neurosci 2023; 26(8): 1449-60.

22. Jack CR, Jr., Bernstein MA, Fox NC, et al. The Alzheimer's Disease Neuroimaging Initiative (ADNI): MRI methods. J Magn Reson Imaging 2008; 27(4): 685-91.

23. Dincer A, Gordon BA, Hari-Raj A, et al. Comparing cortical signatures of atrophy between late-onset and autosomal dominant Alzheimer disease. Neuroimage Clin 2020; 28: 102491.

24. Bashyam VM, Erus G, Doshi J, et al. MRI signatures of brain age and disease over the lifespan based on a deep brain network and 14 468 individuals worldwide. Brain 2020; 143(7): 2312-24.

25. Fortin JP, Parker D, Tunc B, et al. Harmonization of multi-site diffusion tensor imaging data. Neuroimage 2017; 161: 149-70.

26. Fortin JP, Cullen N, Sheline YI, et al. Harmonization of cortical thickness measurements across scanners and sites. Neuroimage 2018; 167: 104-20.

27. Cole JH, Ritchie SJ, Bastin ME, et al. Brain age predicts mortality. Mol Psychiatry 2018; 23(5): 1385-92.

28. de Lange AG, Cole JH. Commentary: Correction procedures in brain-age prediction. Neuroimage Clin 2020; 26: 102229.

29. Le TT, Kuplicki RT, McKinney BA, et al. A Nonlinear Simulation Framework Supports Adjusting for Age When Analyzing BrainAGE. Front Aging Neurosci 2018; 10: 317.

30. Millar PR, Gordon BA, Luckett PH, et al. Multimodal brain age estimates relate to Alzheimer disease biomarkers and cognition in early stages: a cross-sectional observational study. Elife 2023; 12.

31. Lee H, Fenster RJ, Pineda SS, et al. Cell Type-Specific Transcriptomics Reveals that Mutant Huntingtin Leads to Mitochondrial RNA Release and Neuronal Innate Immune Activation. Neuron 2020; 107(5): 891-908 e8.

32. Grubman A, Chew G, Ouyang JF, et al. A single-cell atlas of entorhinal cortex from individuals with Alzheimer's disease reveals cell-type-specific gene expression regulation. Nat Neurosci 2019; 22(12): 2087-97.

33. Zhou Y, Tada M, Cai Z, et al. Human early-onset dementia caused by DAP12 deficiency reveals a unique signature of dysregulated microglia. Nat Immunol 2023; 24(3): 545-57.

6. Acknowledgements:

DIAN Acknowledgement

Data collection and sharing for this project was supported by The Dominantly Inherited Alzheimer’s Network (DIAN, UF1AG032438) funded by the National Institute on Aging (NIA), the German Center for Neurodegenerative Diseases (DZNE), Raul Carrea Institute for Neurological Research (FLENI), Partial support by the Research and Development Grants for Dementia from Japan Agency for Medical Research and Development, AMED, and the Korea Health Technology R&D Project through the Korea Health Industry Development Institute (KHIDI).This manuscript has been reviewed by DIAN Study investigators for scientific content and consistency of data interpretation with previous DIAN Study publications. We acknowledge the altruism of the participants and their families and contributions of the DIAN research and support staff at each of the participating sites for their contributions to this study.

Acknowledgments for the use of ADSP WES and WGS data

The Alzheimer’s Disease Sequencing Project (ADSP) is comprised of two Alzheimer’s Disease (AD) genetics consortia and three National Human Genome Research Institute (NHGRI) funded Large Scale Sequencing and Analysis Centers (LSAC). The two AD genetics consortia are the Alzheimer’s Disease Genetics Consortium (ADGC) funded by NIA (U01 AG032984), and the Cohorts for Heart and Aging Research in Genomic Epidemiology (CHARGE) funded by NIA (R01 AG033193), the National Heart, Lung, and Blood Institute (NHLBI), other National Institute of Health (NIH) institutes and other foreign governmental and non-governmental organizations. The Discovery Phase analysis of sequence data is supported through UF1AG047133 (to Drs. Schellenberg, Farrer, Pericak-Vance, Mayeux, and Haines); U01AG049505 to Dr. Seshadri; U01AG049506 to Dr. Boerwinkle; U01AG049507 to Dr. Wijsman; and U01AG049508 to Dr. Goate and the Discovery Extension Phase analysis is supported through U01AG052411 to Dr. Goate, U01AG052410 to Dr. Pericak-Vance and U01 AG052409 to Drs. Seshadri and Fornage.

Sequencing for the Follow Up Study (FUS) is supported through U01AG057659 (to Drs. PericakVance, Mayeux, and Vardarajan) and U01AG062943 (to Drs. Pericak-Vance and Mayeux). Data generation and harmonization in the Follow-up Phase is supported by U54AG052427 (to Drs. Schellenberg and Wang). The FUS Phase analysis of sequence data is supported through U01AG058589 (to Drs. Destefano, Boerwinkle, De Jager, Fornage, Seshadri, and Wijsman), U01AG058654 (to Drs. Haines, Bush, Farrer, Martin, and Pericak-Vance), U01AG058635 (to Dr. Goate), RF1AG058066 (to Drs. Haines, Pericak-Vance, and Scott), RF1AG057519 (to Drs. Farrer and Jun), R01AG048927 (to Dr. Farrer), and RF1AG054074 (to Drs. Pericak-Vance and Beecham).

The ADGC cohorts include: Adult Changes in Thought (ACT) (UO1 AG006781, UO1 HG004610, UO1 HG006375, U01 HG008657), the Alzheimer’s Disease Centers (ADC) (P30 AG019610, P30 AG013846, P50 AG008702, P50 AG025688, P50 AG047266, P30 AG010133, P50 AG005146, P50 AG005134, P50 AG016574, P50 AG005138, P30 AG008051, P30 AG013854, P30 AG008017, P30 AG010161, P50 AG047366, P30 AG010129, P50 AG016573, P50 AG016570, P50 AG005131, P50 AG023501, P30 AG035982, P30 AG028383, P30 AG010124, P50 AG005133, P50 AG005142, P30 AG012300, P50 AG005136, P50 AG033514, P50 AG005681, and P50 AG047270), the Chicago Health and Aging Project (CHAP) (R01 AG11101, RC4 AG039085, K23 AG030944), Indianapolis Ibadan (R01 AG009956, P30 AG010133), the Memory and Aging Project (MAP) (R01 AG17917), Mayo Clinic (MAYO) (R01 AG032990, U01 AG046139, R01 NS080820, RF1 AG051504, P50 AG016574), Mayo Parkinson’s Disease controls (NS039764, NS071674, 5RC2HG005605), University of Miami (R01 AG027944, R01 AG028786, R01 AG019085, IIRG09133827, A2011048), the Multi-Institutional Research in Alzheimer’s Genetic Epidemiology Study (MIRAGE) (R01 AG09029, R01 AG025259), the National Cell Repository for Alzheimer’s Disease (NCRAD) (U24 AG21886), the National Institute on Aging Late Onset Alzheimer's Disease Family Study (NIA- LOAD) (R01 AG041797), the Religious Orders Study (ROS) (P30 AG10161, R01 AG15819), the Texas Alzheimer’s Research and Care Consortium (TARCC) (funded by the Darrell K Royal Texas Alzheimer's Initiative), Vanderbilt University/Case Western Reserve University (VAN/CWRU) (R01 AG019757, R01 AG021547, R01 AG027944, R01 AG028786, P01 NS026630, and Alzheimer’s Association), the Washington Heights-Inwood Columbia Aging Project (WHICAP) (RF1 AG054023), the University of Washington Families (VA Research Merit Grant, NIA: P50AG005136, R01AG041797, NINDS: R01NS069719), the Columbia University HispanicEstudio Familiar de Influencia Genetica de Alzheimer (EFIGA)

(RF1 AG015473), the University of Toronto (UT) (funded by Wellcome Trust, Medical Research Council, Canadian Institutes of Health Research), and Genetic Differences (GD) (R01 AG007584). The CHARGE cohorts are supported in part by National Heart, Lung, and Blood Institute (NHLBI) infrastructure grant HL105756 (Psaty), RC2HL102419 (Boerwinkle) and the neurology working group is supported by the National Institute on Aging (NIA) R01 grant AG033193.

The CHARGE cohorts participating in the ADSP include the following: Austrian Stroke Prevention Study (ASPS), ASPS-Family study, and the Prospective Dementia Registry-Austria (ASPS/PRODEM-Aus), the Atherosclerosis Risk in Communities (ARIC) Study, the Cardiovascular Health Study (CHS), the Erasmus Rucphen Family Study (ERF), the Framingham Heart Study (FHS), and the Rotterdam Study (RS). ASPS is funded by the Austrian Science Fond (FWF) grant number P20545-P05 and P13180 and the Medical University of Graz. The ASPS-Fam is funded by the Austrian Science Fund (FWF) project I904),the EU Joint Programme - Neurodegenerative Disease Research (JPND) in frame of the BRIDGET project (Austria, Ministry of Science) and the Medical University of Graz and the Steiermärkische Krankenanstalten Gesellschaft. PRODEM-Austria is supported by the Austrian Research Promotion agency (FFG) (Project No. 827462) and by the Austrian National Bank (Anniversary Fund, project 15435. ARIC research is carried out as a collaborative study supported by NHLBI contracts (HHSN268201100005C, HHSN268201100006C, HHSN268201100007C, HHSN268201100008C, HHSN268201100009C, HHSN268201100010C, HHSN268201100011C, and HHSN268201100012C). Neurocognitive data in ARIC is collected by U01 2U01HL096812, 2U01HL096814, 2U01HL096899, 2U01HL096902, 2U01HL096917 from the NIH (NHLBI, NINDS, NIA and NIDCD), and with previous brain MRI examinations funded by R01-HL70825 from the NHLBI. CHS research was supported by contracts HHSN268201200036C, HHSN268200800007C, N01HC55222, N01HC85079, N01HC85080, N01HC85081, N01HC85082, N01HC85083, N01HC85086, and grants U01HL080295 and U01HL130114 from the NHLBI with additional contribution from the National Institute of Neurological Disorders and Stroke (NINDS). Additional support was provided by R01AG023629, R01AG15928, and R01AG20098 from the NIA. FHS research is supported by NHLBI contracts N01-HC-25195 and HHSN268201500001I. This study was also supported by additional grants from the NIA (R01s AG054076, AG049607 and AG033040 and NINDS (R01 NS017950). The ERF study as a part of EUROSPAN (European Special Populations Research Network) was supported by European Commission FP6 STRP grant number 018947 (LSHG-CT-2006-01947) and also received funding from the European Community's Seventh Framework Programme (FP7/2007-2013)/grant agreement HEALTH-F4- 2007-201413 by the European Commission under the programme "Quality of Life and Management of the Living Resources" of 5th Framework Programme (no. QLG2-CT-2002- 01254). High-throughput analysis of the ERF data was supported by a joint grant from the Netherlands Organization for Scientific Research and the Russian Foundation for Basic Research (NWO-RFBR 047.017.043). The Rotterdam Study is funded by Erasmus Medical Center and Erasmus University, Rotterdam, the Netherlands Organization for Health Research and Development (ZonMw), the Research Institute for Diseases in the Elderly (RIDE), the Ministry of Education, Culture and Science, the Ministry for Health, Welfare and Sports, the European Commission (DG XII), and the municipality of Rotterdam. Genetic data sets are also supported by the Netherlands Organization of Scientific Research NWO Investments (175.010.2005.011, 911-03-012), the Genetic Laboratory of the Department of Internal Medicine, Erasmus MC, the Research Institute for Diseases in the Elderly (014-93-015; RIDE2), and the Netherlands Genomics Initiative (NGI)/Netherlands Organization for Scientific Research (NWO) Netherlands Consortium for Healthy Aging (NCHA), project 050-060-810. All studies are grateful to their participants, faculty and staff. The content of these manuscripts is solely the responsibility of the authors and does not necessarily represent the official views of the National Institutes of Health or the U.S. Department of Health and Human Services.

The FUS cohorts include: the Alzheimer’s Disease Centers (ADC) (P30 AG019610, P30 AG013846, P50 AG008702, P50 AG025688, P50 AG047266, P30 AG010133, P50 AG005146, P50 AG005134, P50 AG016574, P50 AG005138, P30 AG008051, P30 AG013854, P30 AG008017, P30 AG010161, P50 AG047366, P30 AG010129, P50 AG016573, P50 AG016570, P50 AG005131, P50 AG023501, P30 AG035982, P30 AG028383, P30 AG010124, P50 AG005133, P50 AG005142, P30 AG012300, P50 AG005136, P50 AG033514, P50 AG005681, and P50 AG047270), Alzheimer's Disease Neuroimaging Initiative (ADNI) (U19AG024904), Amish Protective Variant Study (RF1AG058066), Cache County Study (R01AG11380, R01AG031272, R01AG21136, RF1AG054052), Case Western Reserve University Brain Bank (CWRUBB) (P50AG008012), Case Western Reserve University Rapid Decline (CWRURD) (RF1AG058267, NU38CK000480), CubanAmerican Alzheimer's Disease Initiative (CuAADI) (3U01AG052410),

Estudio Familiar de Influencia Genetica en Alzheimer (EFIGA) (5R37AG015473, RF1AG015473, R56AG051876), Genetic and Environmental Risk Factors for Alzheimer Disease Among African Americans Study (GenerAAtions) (2R01AG09029, R01AG025259, 2R01AG048927), Gwangju Alzheimer and Related Dementias Study (GARD) (U01AG062602), Hussman Institute for Human Genomics Brain Bank (HIHGBB) (R01AG027944, Alzheimer's Association "Identification of Rare Variants in Alzheimer Disease"), Ibadan Study of Aging (IBADAN) (5R01AG009956), Mexican Health and Aging Study (MHAS) (R01AG018016), Multi-Institutional Research in Alzheimer's Genetic Epidemiology (MIRAGE) (2R01AG09029, R01AG025259, 2R01AG048927), Northern Manhattan Study (NOMAS) (R01NS29993), Peru Alzheimer's Disease Initiative (PeADI) (RF1AG054074), Puerto Rican 1066 (PR1066) (Wellcome Trust (GR066133/GR080002), European Research Council (340755)), Puerto Rican Alzheimer Disease Initiative (PRADI) (RF1AG054074), Reasons for Geographic and Racial Differences in Stroke (REGARDS) (U01NS041588), Research in African American Alzheimer Disease Initiative (REAAADI) (U01AG052410), Rush Alzheimer's Disease Center (ROSMAP) (P30AG10161, R01AG15819, R01AG17919), University of Miami Brain Endowment Bank (MBB), and University of Miami/Case Western/North Carolina A&T African American (UM/CASE/NCAT) (U01AG052410, R01AG028786).

The four LSACs are: the Human Genome Sequencing Center at the Baylor College of Medicine (U54 HG003273), the Broad Institute Genome Center (U54HG003067), The American Genome Center at the Uniformed Services University of the Health Sciences (U01AG057659), and the Washington University Genome Institute (U54HG003079).

Biological samples and associated phenotypic data used in primary data analyses were stored at Study Investigators institutions, and at the National Cell Repository for Alzheimer's Disease (NCRAD, U24AG021886) at Indiana University funded by NIA. Associated Phenotypic Data used in primary and secondary data analyses were provided by Study Investigators, the NIA funded Alzheimer's Disease Centers (ADCs), and the National Alzheimer's Coordinating Center (NACC, U01AG016976) and the National Institute on Aging Genetics of Alzheimer's Disease Data Storage Site (NIAGADS, U24AG041689) at the University of Pennsylvania, funded by NIA This research was supported in part by the Intramural Research Program of the National Institutes of health, National Library of Medicine. Contributors to the Genetic Analysis Data included Study Investigators on projects that were individually funded by NIA, and other NIH institutes, and by private U.S. organizations, or foreign governmental or nongovernmental organizations.

An up-to-date acknowledgment statement can be found on the ADSP site: https://www.niagads.org/adsp/content/acknowledgement-statement.

Data collection and sharing for this project was funded by the Alzheimer's Disease Neuroimaging Initiative (ADNI) (National Institutes of Health Grant U01 AG024904) and DOD ADNI (Department of Defense award number W81XWH-12-2-0012). ADNI is funded by the National Institute on Aging, the National Institute of Biomedical Imaging and Bioengineering, and through generous contributions from the following: AbbVie, Alzheimer's Association; Alzheimer's Drug Discovery Foundation; Araclon Biotech; BioClinica, Inc.; Biogen; Bristol-Myers Squibb Company; CereSpir, Inc.; Cogstate; Eisai Inc.; Elan Pharmaceuticals, Inc.; Eli Lilly and Company; EuroImmun; F. Hoffmann-La Roche Ltd and its affiliated company Genentech, Inc.; Fujirebio; GE Healthcare; IXICO Ltd.; Janssen Alzheimer Immunotherapy Research & Development, LLC.; Johnson & Johnson Pharmaceutical Research & Development LLC.; Lumosity; Lundbeck; Merck & Co., Inc.; Meso Scale Diagnostics, LLC.; NeuroRx Research; Neurotrack Technologies; Novartis Pharmaceuticals Corporation; Pfizer Inc.; Piramal Imaging; Servier; Takeda Pharmaceutical Company; and Transition Therapeutics. The Canadian Institutes of Health Research is providing funds to support ADNI clinical sites in Canada. Private sector contributions are facilitated by the Foundation for the National Institutes of Health (www.fnih.org). The grantee organization is the Northern California Institute for Research and Education, and the study is coordinated by the Alzheimer's Therapeutic Research Institute at the University of Southern California. ADNI data are disseminated by the Laboratory for Neuro Imaging at the University of Southern California.

Additional information to include in an acknowledgment statement can be found on the LONI site: https://adni.loni.usc.edu/wp-content/uploads/how_to_apply/ADNI_Data_Use_Agreement.pdf.

The Alzheimer's Disease Genetics Consortium (ADGC) supported sample preparation, whole exome sequencing and data processing through NIA grant U01AG032984. Sequencing data generation and harmonization is supported by the Genome Center for Alzheimer's Disease, U54AG052427, and data sharing is supported by NIAGADS, U24AG041689. Samples from the National Centralized Repository for Alzheimer's Disease and Related Dementias (NCRAD), which receives government support under a cooperative agreement grant (U24 AG021886) awarded by the National Institute on Aging (NIA), were used in this study. We thank contributors who collected samples used in this study, as well as patients and their families, whose help and participation made this work possible. NIH grants supported enrollment and data collection for the individual studies including: GenerAAtions R01AG20688 (PI M. Daniele Fallin, PhD); Miami/Duke R01 AG027944, R01 AG028786 (PI Margaret A. Pericak-Vance, PhD); NC A&T P20 MD000546, R01 AG28786-01A1 (PI Goldie S. Byrd, PhD); Case Western (PI Jonathan L. Haines, PhD); MIRAGE R01 AG009029 (PI Lindsay A. Farrer, PhD); ROS P30AG10161, R01AG15819, R01AG30146, TGen (PI David A. Bennett, MD); MAP R01AG17917, R01AG15819, TGen (PI David A. Bennett, MD). The NACC database is funded by NIA/NIH Grant U01 AG016976. NACC data are contributed by the NIA-funded ADCs: P30 AG019610 (PI Eric Reiman, MD), P30 AG013846 (PI Neil Kowall, MD), P30 AG062428-01 (PI James Leverenz, MD) P50 AG008702 (PI Scott Small, MD), P50 AG025688 (PI Allan Levey, MD, PhD), P50 AG047266 (PI Todd Golde, MD, PhD), P30 AG010133 (PI Andrew Saykin, PsyD), P50 AG005146 (PI Marilyn Albert, PhD), P30 AG062421-01 (PI Bradley Hyman, MD, PhD), P30 AG062422-01 (PI Ronald Petersen, MD, PhD), P50 AG005138 (PI Mary Sano, PhD), P30 AG008051 (PI Thomas Wisniewski, MD), P30 AG013854 (PI Robert Vassar, PhD), P30 AG008017 (PI Jeffrey Kaye, MD), P30 AG010161 (PI David Bennett, MD), P50 AG047366 (PI Victor Henderson, MD, MS), P30 AG010129 (PI Charles DeCarli, MD), P50 AG016573 (PI Frank LaFerla, PhD), P30 AG062429-01(PI James Brewer, MD, PhD), P50 AG023501 (PI Bruce Miller, MD), P30 AG035982 (PI Russell Swerdlow, MD), P30 AG028383 (PI Linda Van Eldik, PhD), P30 AG053760 (PI Henry Paulson, MD, PhD), P30 AG010124 (PI John Trojanowski, MD, PhD), P50 AG005133 (PI Oscar Lopez, MD), P50 AG005142 (PI Helena Chui, MD), P30 AG012300 (PI Roger Rosenberg, MD), P30 AG049638 (PI Suzanne Craft, PhD), P50 AG005136 (PI Thomas Grabowski, MD), P30 AG062715-01 (PI Sanjay Asthana, MD, FRCP), P50 AG005681 (PI John Morris, MD), P50 AG047270 (PI Stephen Strittmatter, MD, PhD).

This work was supported by grants from the National Institutes of Health (R01AG044546, P01AG003991, RF1AG053303, R01AG058501, U01AG058922, RF1AG058501 and R01AG057777). The recruitment and clinical characterization of research participants at Washington University were supported by NIH P50 AG05681, P01 AG03991, and P01 AG026276. This work was supported by access to equipment made possible by the Hope Center for Neurological Disorders, and the Departments of Neurology and Psychiatry at Washington University School of Medicine.

We thank the contributors who collected samples used in this study, as well as patients and their families, whose help and participation made this work possible. Members of the National Institute on Aging Late-Onset Alzheimer Disease/National Cell Repository for Alzheimer Disease (NIA-LOAD NCRAD) Family Study Group include the following: Richard Mayeux, MD, MSc; Martin Farlow, MD; Tatiana Foroud, PhD; Kelley Faber, MS; Bradley F. Boeve, MD; Neill R. Graff-Radford, MD; David A. Bennett, MD; Robert A. Sweet, MD; Roger Rosenberg, MD; Thomas D. Bird, MD; Carlos Cruchaga, PhD; and Jeremy M. Silverman, PhD.

This work was partially supported by grant funding from NIH R01 AG039700 and NIH P50 AG005136. Subjects and samples used here were originally collected with grant funding from NIH U24 AG026395, U24 AG021886, P50 AG008702, P01 AG007232, R37 AG015473, P30 AG028377, P50 AG05128, P50 AG16574, P30 AG010133, P50 AG005681, P01 AG003991, U01MH046281, U01 MH046290 and U01 MH046373. The funders had no role in study design, analysis or preparation of the manuscript. The authors declare no competing interests.

This work was supported by the National Institutes of Health (R01 AG027944, R01 AG028786 to MAPV, R01 AG019085 to JLH, P20 MD000546); a joint grant from the Alzheimer's Association (SG-14-312644) and the Fidelity Biosciences Research Initiative to MAPV; the BrightFocus Foundation (A2011048 to MAPV). NIA-LOAD Family-Based Study supported the collection of samples used in this study through NIH grants U24 AG026395 and R01 AG041797 and the MIRAGE cohort was supported through the NIH grants R01 AG025259 and R01 AG048927. We thank contributors, including the Alzheimer's disease Centers who collected samples used in this study, as well as

patients and their families, whose help and participation made this work possible. Study design: HNC, BWK, JLH, MAPV; Sample collection: MLC, JMV, RMC, LAF, JLH, MAPV; Whole exome sequencing and Sanger sequencing: SR, PLW; Sequencing data analysis: HNC, BWK, KLHN, SR, MAK, JRG, ERM, GWB, MAPV; Statistical analysis: BWK, KLHN, JMJ, MAPV; Preparation of manuscript: HNC, BWK. The authors jointly discussed the experimental results throughout the duration of the study. All authors read and approved the final manuscript.

Data collection and sharing for this project was supported by the Washington Heights-Inwood Columbia Aging Project (WHICAP, PO1AG07232, R01AG037212, RF1AG054023) funded by the National Institute on Aging (NIA) and by the National Center for Advancing Translational Sciences, National Institutes of Health, through Grant Number UL1TR001873. This manuscript has been reviewed by WHICAP investigators for scientific content and consistency of data interpretation with previous WHICAP Study publications. We acknowledge the WHICAP study participants and the WHICAP research and support staff for their contributions to this study.

This work was supported by grants from the National Institutes of Health (R01AG044546, P01AG003991, RF1AG053303, R01AG058501, U01AG058922, RF1AG058501 and R01AG057777). The recruitment and clinical characterization of research participants at Washington University were supported by NIH P50 AG05681, P01 AG03991, and P01 AG026276. This work was supported by access to equipment made possible by the Hope Center for Neurological Disorders, and the Departments of Neurology and Psychiatry at Washington University School of Medicine.

We thank the contributors who collected samples used in this study, as well as patients and their families, whose help and participation made this work possible. Members of the National Institute on Aging Late-Onset Alzheimer Disease/National Cell Repository for Alzheimer Disease (NIA-LOAD NCRAD) Family Study Group include the following: Richard Mayeux, MD, MSc; Martin Farlow, MD; Tatiana Foroud, PhD; Kelley Faber, MS; Bradley F. Boeve, MD; Neill R. Graff-Radford, MD; David A. Bennett, MD; Robert A. Sweet, MD; Roger Rosenberg, MD; Thomas D. Bird, MD; Carlos Cruchaga, PhD; and Jeremy M. Silverman, PhD.

This work was supported by grants from the National Institutes of Health (R01AG044546, P01AG003991, RF1AG053303, R01AG058501, U01AG058922, RF1AG058501 and R01AG057777). The recruitment and clinical characterization of research participants at Washington University were supported by NIH P50 AG05681, P01 AG03991, and P01 AG026276. This work was supported by access to equipment made possible by the Hope Center for Neurological Disorders, and the Departments of Neurology and Psychiatry at Washington University School of Medicine.

We thank the contributors who collected samples used in this study, as well as patients and their families, whose help and participation made this work possible. Members of the National Institute on Aging Late-Onset Alzheimer Disease/National Cell Repository for Alzheimer Disease (NIA-LOAD NCRAD) Family Study Group include the following: Richard Mayeux, MD, MSc; Martin Farlow, MD; Tatiana Foroud, PhD; Kelley Faber, MS; Bradley F. Boeve, MD; Neill R. Graff-Radford, MD; David A. Bennett, MD; Robert A. Sweet, MD; Roger Rosenberg, MD; Thomas D. Bird, MD; Carlos Cruchaga, PhD; and Jeremy M. Silverman, PhD.

Mayo RNAseq Study- Study data were provided by the following sources: The Mayo Clinic Alzheimer's Disease Genetic Studies, led by Dr. Nilufer Ertekin-Taner and Dr. Steven G. Younkin, Mayo Clinic, Jacksonville, FL using samples from the Mayo Clinic Study of Aging, the Mayo Clinic Alzheimer's Disease Research Center, and the Mayo Clinic Brain Bank. Data collection was supported through funding by NIA grants P50 AG016574, R01 AG032990, U01 AG046139, R01 AG018023, U01 AG006576, U01 AG006786, R01 AG025711, R01 AG017216, R01 AG003949, NINDS grant R01 NS080820, CurePSP Foundation, and support from Mayo Foundation. Study data includes samples collected through the Sun Health Research Institute Brain and Body Donation Program of Sun City, Arizona. The Brain and Body Donation Program is supported by the National Institute of Neurological Disorders and Stroke (U24 NS072026 National Brain and Tissue Resource for Parkinson's Disease and Related Disorders), the National Institute on Aging (P30 AG19610 Arizona Alzheimer's Disease Core Center), the Arizona Department of Health Services (contract 211002, Arizona Alzheimer's Research Center), the Arizona Biomedical Research

Commission (contracts 4001, 0011, 05-901 and 1001 to the Arizona Parkinson's Disease Consortium) and the Michael J. Fox Foundation for Parkinson's Research

ROSMAP- We are grateful to the participants in the Religious Order Study, the Memory and Aging Project. This work is supported by the US National Institutes of Health [U01 AG046152, R01 AG043617, R01 AG042210, R01 AG036042, R01 AG036836, R01 AG032990, R01 AG18023, RC2 AG036547, P50 AG016574, U01 ES017155, KL2 RR024151, K25 AG041906-01, R01 AG30146, P30 AG10161, R01 AG17917, R01 AG15819, K08 AG034290, P30 AG10161 and R01 AG11101.

Mount Sinai Brain Bank (MSBB)- This work was supported by the grants R01AG046170, RF1AG054014, RF1AG057440 and R01AG057907 from the NIH/National Institute on Aging (NIA). R01AG046170 is a component of the AMP-AD Target Discovery and Preclinical Validation Project. Brain tissue collection and characterization was supported by NIH HHSN271201300031C.

This study was supported by the National Institute on Aging (NIA) grants AG030653, AG041718, AG064877 and P30-AG066468.

We would like to thank study participants, their families, and the sample collectors for their invaluable contributions. This research was supported in part by the National Institute on Aging grant U01AG049508 (PI Alison M. Goate). This research was supported in part by Genentech, Inc. (PI Alison M. Goate, Robert R. Graham).

The NACC database is funded by NIA/NIH Grant U01 AG016976. NACC data are contributed by these NIA-funded ADCs: P30 AG013846 (PI Neil Kowall, MD), P50 AG008702 (PI Scott Small, MD), P50 AG025688 (PI Allan Levey, MD, PhD), P30 AG010133 (PI Andrew Saykin, PsyD), P50 AG005146 (PI Marilyn Albert, PhD), P50 AG005134 (PI Bradley Hyman, MD, PhD), P50 AG016574 (PI Ronald Petersen, MD, PhD), P30 AG013854 (PI M. Marsel Mesulam, MD), P30 AG008017 (PI Jeffrey Kaye, MD), P30 AG010161 (PI David Bennett, MD), P30 AG010129 (PI Charles DeCarli, MD), P50 AG016573 (PI Frank LaFerla, PhD), P50 AG005131 (PI Douglas Galasko, MD), P30 AG028383 (PI Linda Van Eldik, PhD), P30 AG010124 (PI John Trojanowski, MD, PhD), P50 AG005142 (PI Helena Chui, MD), P30 AG012300 (PI Roger Rosenberg, MD), P50 AG005136 (PI Thomas Grabowski, MD), P50 AG005681 (PI John Morris, MD), P30 AG028377 (Kathleen Welsh-Bohmer, PhD), and P50 AG008671 (PI Henry Paulson, MD, PhD).

Samples from the National Cell Repository for Alzheimer’s Disease (NCRAD), which receives government support under a cooperative agreement grant (U24 AG21886) awarded by the National Institute on Aging (NIA), were used in this study. We thank contributors who collected samples used in this study, as well as patients and their families, whose help and participation made this work possible.

The Alzheimer's Disease Genetics Consortium supported the collection of samples used in this study through National Institute on Aging (NIA) grants U01AG032984 and RC2AG036528.

We acknowledge the generous contributions of the Cache County Memory Study participants. Sequencing for this study was funded by RF1AG054052 (PI: John S.K. Kauwe).
